# Tracking Lithium Intercalation in Battery Electrodes via Their Electrochromic Properties Using *Operando* Ellipsometry

**DOI:** 10.1021/jacs.5c14539

**Published:** 2025-10-21

**Authors:** Jialin Gu, Adam J. Lovett, Máté Füredi, Thomas Dore, Stefan Guldin, Thomas S. Miller

**Affiliations:** † Department of Chemical Engineering, 4919 University College London, Torrington Place, London WC1E 7JE, United Kingdom; ‡ Advanced Propulsion Lab, Marshgate, University College London, London E20 2AE, United Kingdom; § The Faraday Institution, Quad One, Didcot OX11 ORA, United Kingdom; ∥ Semilab Co. Ltd., Prielle Kornélia u. 2, Budapest H-1117, Hungary; ⊥ Department of Life Science Engineering, Technical University of Munich, Freising 85354, Germany; # TUMCREATE, 1 CREATE Way, #10-02 CREATE Tower, 138602, Singapore

## Abstract

A deep understanding of charge storage mechanisms in lithium-ion battery electrodes is necessary to advance next-generation materials for fast charging and high-power-density applications. Under high-rate conditions, disentangling charge storage contributions (e.g., intercalation, alloying, pseudocapacitance, capacitance, interphase formation, electrolyte degradation, and other processes) is complex, especially for thin film and nanosized materials that typically exhibit surface-dominated behavior. Often overlooked, the electrochromic properties of lithium-ion battery electrodes are innately related to their charge storage properties. In this work, we demonstrate how *operando* ellipsometry provides detailed insights into charge storage kinetics in battery electrodes. Using a model TiO_2_-anatase thin film, we validate the energy loss function (ELF) derived from the complex dielectric constant as a key indicator for tracking the lithiation state and thus state of charge. Further, we demonstrate that d­(ELF)/dV acts as an “opto-voltammogram” that can be used to selectively probe and deconvolute redox reactions, analogous to differential capacity analysis (dQ/dV) or cyclic voltammetry. These findings establish spectroscopic ellipsometry as a powerful technique for optimizing materials in energy storage applications.

## Graphical abstract

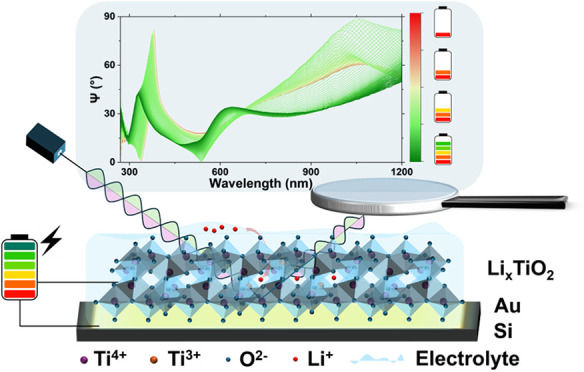

## Introduction

Understanding the mechanisms of charge storage in battery materials is crucial to enable advancement of their performance and reliability.^1,2^ However, for novel materials, these mechanisms remain elusive due to the complex interplay of electrochemical, structural, and interfacial processes, which current analytical techniques struggle to fully capture. In recent years, *operando* optical tools have gained renewed interest due to their high resolution, nondestructive nature, and ease of operation (i.e., nonionizing light sources).^3–6^ Yet, despite these benefits, optical microscopy is constrained by its surface-specific sensitivity,^3,4,7^ while fluorescence interference and the presence of electrolytes complicate data analysis in Raman spectroscopy.^8^ In this context, spectroscopic ellipsometry (SE) has recently emerged as a promising substitute for studying thin-film lithium-ion batteries (LIBs) under electrochemical control.^9–12^ Yet, to date, the interpretation of ellipsometric signals during electrochemical cycling lacks standardization, inhibiting the technique from reaching its full potential. In brief, ellipsometry measures the complex reflectance ratio (ρ)

$$\rho =\frac{r_{p}}{r_{s}}=\tan(\psi )\cdot e^{-i\Delta }$$ 1

given by the ratio of Fresnel-reflection coefficients corresponding to light polarized parallel (*p*-plane) and perpendicular (*s*-plane) to the plane of incidence, respectively, *r*_*p*_ and *r*_*s*_. Within these, ψ describes how the amplitude ratio changes after reflection and Δ describes the phase shift between the components. By modeling the elliptically polarized reflected response, key optical properties such as refractive index (*n*), extinction coefficient (*k*), film thickness (*t*), and complex dielectric function (ε) can be determined as a function of wavelength (λ).^13,14^ In turn, these optical properties are related to key properties of the thin film, including composition, crystallinity, film thickness, porosity, and roughness,^14,15^ enabling a wealth of information to be characterized with high sensitivity.

The complex dielectric function, ε­(λ), is strongly influenced by electronic transitions between valence and conduction band states, particularly in the optical frequency range (ultraviolet-visible-infrared).^16^ Through the analysis of ε­(λ),^17,18^ behavior related to the optical properties of the material can be investigated in detail, including oscillator contributions and the nature of electronic states near the band edges. Thus, by monitoring the band gap via *operando* ellipsometry (OE), we can monitor the state of charge (SOC) of the material in real time. Several LIB-relevant oxides display electrochromic properties while under electrochemical control during lithiation, inducing a change in the band structure and the oxidation state of a redox-active species within.^11,12,19–21^ When this change occurs in the typical SE wavelength range (∼200–2000 nm, 0.6–6 eV), electrochemical charge storage can be directly tracked from the electrochromic response of the material in an *operando* manner, independent of the current response. Crucially, by monitoring a characteristic wavelength which is specific to the transition energy related to the redox-active species, we can selectively tune the response to filter out the contributions unrelated to charge storage. This is particularly advantageous as thin films often exhibit pseudocapacitive behavior (broadened redox features and loss of plateauing in charge-discharge curves).^22^ Strictly, pseudocapacitance is a reversible Faradaic redox process (i.e., charge transfer reaction) that occurs at or near the surface of a material while under electrochemical control, or when these reactions are not under diffusion control.^23^ While the ν/ν^1/2^ scan rate diagnosis approach (also known as Dunn’s method) has become a general method to quantify pseudocapacitance from cyclic voltammetry data,^22,24^ it is often incorrectly applied leading to misinterpretations, and in some cases, inappropriate conclusions, about the nature of charge storage.^25^ Therefore, it is highly relevant to devise a set of independent optically derived parameters that correlate with electrochemical responses of the material (i.e., SOC, dQ/dV, etc.).

Herein, we report the evolution of electrochromic properties of TiO_2_-anatase thin films while under electrochemical control at different scan rates with OE. TiO_2_-anatase is a widely studied LIB anode material due to its low cost, relative abundance, structural stability, safety, and higher operating voltage which mitigates Li plating and minimizes solid electrolyte interface (SEI) formation.^26,27^ At the nanoscale, it combines diffusion-controlled Faradaic redox processes with pseudocapacitive charge storage.^28,29^ We show that the OE can be used to monitor the evolution of characteristic peaks in the energy loss function (ELF) spectrum induced by changes in the titanium oxidation state in TiO_2_-anatase films. Thus, this enables real-time evaluation of the lithiation state as a function of scan rate, independent of the current signal collected during cyclic voltammetry (CV). We demonstrate that the derivative d­(ELF)/dV acts as “opto-voltammogram” that can be used to track a specific redox reaction in an analogous way to differential capacity analysis (dQ/dV) or CV. By simultaneous comparison of “opto-voltammograms” and CV data, the Faradaic charge storage contribution can be much more reliably identified. Additionally, we demonstrate that this opto-voltammogram approach can be used to assess kinetic charge storage limitations, circumnavigating the linearity requirement of Dunn’s method (which is often only valid over a narrow range of CV scan rates). Further, not only does OE offer exceptional selectivity and sensitivity to specific Faradaic redox processes within thin-film electrodes, but it also enables the analysis of film thickness evolution in a noncontact way, providing a versatile tool for understanding volumetric changes during cycling.^15^ These findings lay the groundwork for employing OE to investigate charge storage mechanisms in more complex systems, establishing new pathways to optimize materials for improved energy storage, fast charging, and enhanced power density.^2^

## Results

### Structural and Electrochemical Characterization of TiO_2_-Anatase Thin Films

We first consider the structural characterization of TiO_2_-anatase thin films, grown by sol-gel synthesis onto Au-coated silicon wafer (see the Methodology section). Raman spectroscopy mapping (Supporting Information Figure S1) on uncycled films confirms the presence of anatase TiO_2_ phase via the presence of characteristic peaks at 144, 197, 399, 513, and 639 cm^–1^.^30–33^ Both atomic force microscopy (AFM) (Supporting Information Figure S2) and scanning electron microscopy (SEM) (Supporting Information Figure S3) confirm the successful growth of dense, nonporous TiO_2_.

Next, we investigated the electrochemical performance of our TiO_2_-anatase thin films, which form the baseline of our *operando* ellipsometry studies. A CV collected at 0.1 mV s^–1^ during *operando* ellipsometry (Figure 1a) shows the characteristic reversible redox peaks associated with TiO_2_-anatase: a pair of redox peaks at 1.9 V (oxidation) and 1.5 V (reduction) vs Li/Li^+^ via the Ti^3+^/Ti^4+^ redox couple. Lithiation is further confirmed with *ex situ* Raman spectroscopy mapping post-CV (Supporting Information Figure S1b), revealing an additional peak at 230 cm^–1^, which is characteristic of lithiated TiO_2_-anatase.^34,35^

**Figure 1:**
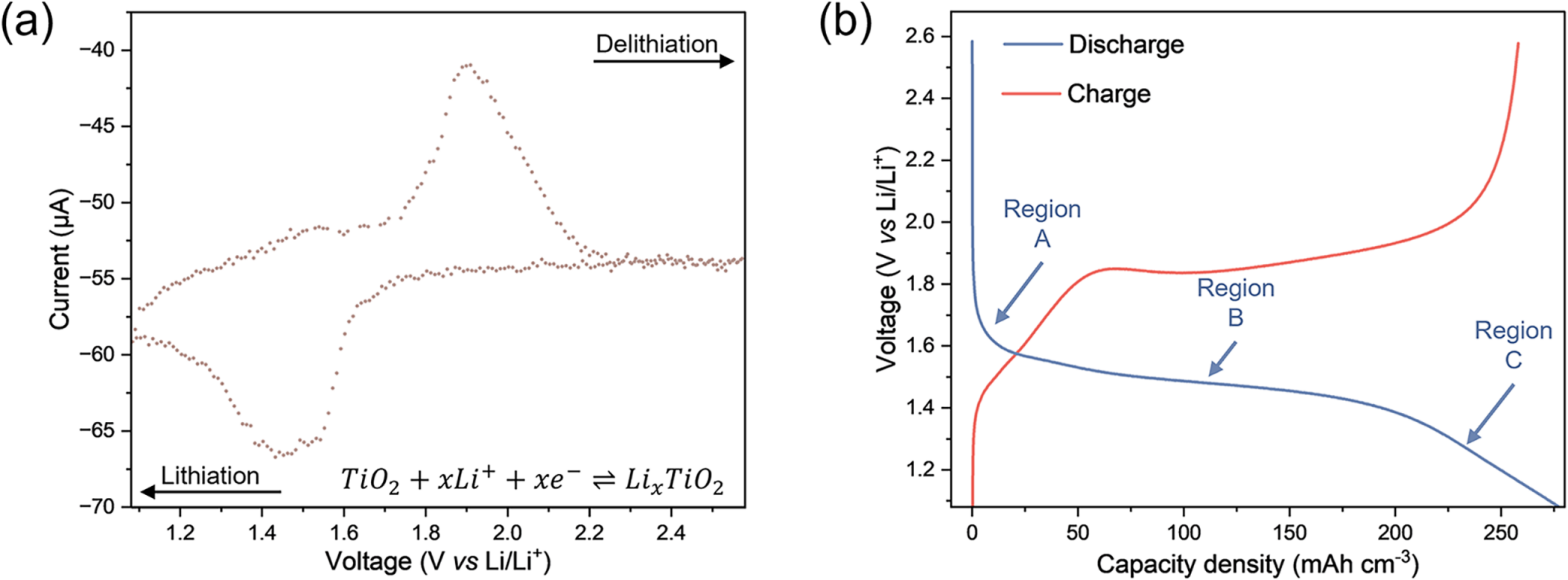
Electrochemical characterization of TiO_2_-anatase thin films. (a) Cyclic voltammogram of the TiO_2_-anatase thin film collected at 0.1 mV s^–1^. A pair of redox peaks are observed, assigned to the reversible lithiation of TiO_2_-anatase. (b) Galvanostatic charge-discharge curve of TiO_2_-anatase collected at 0.9 C. Three distinct features are observed (labeled): Region A (solid solution lithiation), Region B (two-phase region), and Region C (formation of Li-rich tetragonal Li_0.5+x_TiO_2_).

To further validate the electrochemical nature of charge storage, we also closely investigate the features of a characteristic charge-discharge curve collected during galvanostatic cycling at 0.9 C (Figure 1b, where the calculations of C-rates were based on the maximum volumetric capacity of 275 mAh cm^–3^ at 1 C). Three distinct regions are observed (labeled in Figure 1b), consistent with previous electrochemical studies:^35–38^ Region A exhibits a small increase in capacity from the upper cutoff voltage (2.6 V) to approximately 1.6 V, attributed to Li^+^ insertion into the tetragonal anatase lattice (Li_*x*_TiO_2_) without the nucleation of other phases.^35,36^ The amount of Li^+^ insertion achievable in Region A depends on particle size/film thickness,^35^ with smaller particles/thinner films allowing a higher Li^+^ uptake per TiO_2_ formula unit.^38^ For instance, anatase TiO_2_ nanoparticles with a diameter of 196 nm can accommodate Li_0.028_TiO_2_ in this region, whereas nanoparticles with a diameter of 45 nm increase the accommodation to Li_0.05_TiO_2_.^35^ Region B: A plateau at a potential of ∼1.6 V corresponding to the two-phase region containing orthorhombic Li_0.5_TiO_2_ (β-TiO_2_) coexisting with the Li-poor tetragonal Li_*x*_TiO_2_ phases (α-TiO_2_).^35,39–41^ The length of the plateau is dependent on the particle size/film thickness.^35^ Region C: A smooth voltage tail that contains pseudoplateaux from ∼1.4 V *vs* Li/Li^+^ to the lower cutoff voltage (1.08 V) attributed to Li insertion to form the lithium-rich tetragonal Li_0.5+x_TiO_2_. The size of this region increases in nanosized TiO_2_.^22,35,37^

### *Operando* Ellipsometry during Cyclic Voltammetry (ν = 0.1 mV s^–1^)

Next, the variation in electrochromic properties of TiO_2_-anatase films is investigated with *ex situ* ellipsometry (green dashed, Supporting Information Figure S4), *in situ* ellipsometry in 1 M LiClO_4_ in propylene carbonate (PC) electrolyte at open-circuit voltage (OCV) (pink, Supporting Information Figure S4), and while under electrochemical control, with *operando* ellipsometry (Figure 2). By monitoring the evolution of the ellipsometric spectra during cycling, structural information about the film can be determined. The reversible Faradaic lithiation of TiO_2_ anatase has the general equation

$$\mathrm{Ti}O_{2}+xLi^{+}+xe^{-}⇌Li_{x}\mathrm{Ti}O_{2}$$ 2

where titanium is reduced from Ti^4+^ to Ti^3+^ during lithiation. This is accompanied by a change in the electrochromic properties of the TiO_2_-anatase film. Thus, by tracking and fitting variations in the ellipsometric spectra at different voltages (Supporting Information Figure S5), *operando* SE measurements enable the optical properties (n, k) (Supporting Information Figures S6) thickness (Supporting Information Figure S7), and thus composition of the film to be tracked in real time. We fit our ellipsometry data with an optical model (see the Methodology section for further details), the physical significance of which is shown schematically in Figure 2a, inset.

**Figure 2:**
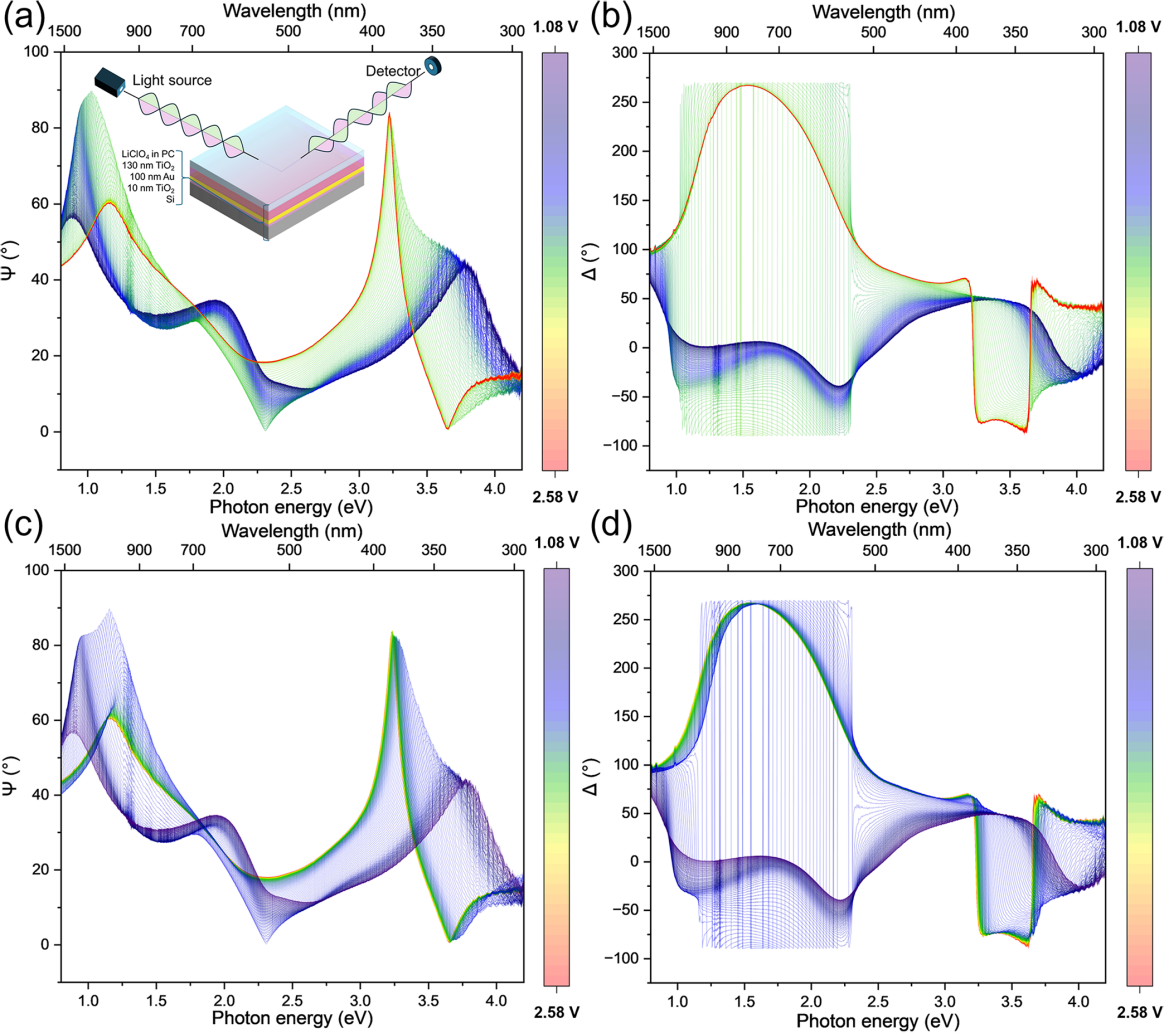
*Operando* spectroscopic ellipsometry characterization of TiO_2_-anatase thin films (a) Ψ and (b) Δ during lithiation plus (c) Ψ and (d) Δ during charging (delithiation). Data was collected during cyclic voltammetry at 0.1 mV s^–1^ (Figure 1a). There is a clear reversible change in the polarized optical signal, concurrent with a change of lithiation state during cycling. The optical model used to fit the raw data is the onset in (a).

*Operando* ellipsometry spectra with a slow CV scan rate of ν = 0.1 mV s^–1^ were studied in detail (Figure 2), which serves as a baseline for further experiments. The evolution of the raw ellipsometric spectra (Ψ, Δ) vs voltage (Li/Li^+^) collected during CV at a scan rate of 0.1 mV s^–1^ is presented in Figure 2a,b (charging) and 2c,d (discharging). Visual inspection of the ψ spectra at 2.58 V vs Li/Li^+^ (Figure 2a, red) reveals peak amplitudes at ∼1.3 and 3.2 eV. After discharge to 1.08 V vs Li/Li^+^ (Figure 2a, blue), these peaks shift to below 1 eV and to ∼3.8 eV, respectively. The Δ spectra (Figure 2b,d) exhibit a substantial change in peak intensity without a significant peak shift. This behavior aligns with the expected lithiation mechanism of anatase TiO_2_, where notable band structure changes occur with a minimal thickness variation. Further, the return of (ψ, Δ) values to their initial values after a full cycling confirms the reversibility of lithium intercalation into TiO_2_-anatase, as expected. Thus, some information can be determined directly from the raw (ψ, Δ) *operando* ellipsometry data, which is mostly related to qualitative conclusions about reversible cyclability. However, a more quantitative analysis requires the optical modeling of the system (i.e., determining the complex refractive index dispersion, *ñ*(λ) = *n*(λ) + *ik*(λ), where *n*(λ) and *k*(λ) represent the refractive index and extinction coefficients, respectively).

Film thickness is also deduced from the optical model (Supporting Figure S7). Initially, at 2.4 V vs Li/Li^+^, the TiO_2_-anatase film has a stable thickness of 132 nm. At 1.4 V (corresponding to the end of the Region B plateau), the thickness increases to 135 nm, a 2.3% increase compared to the initial thickness at 2.4 V. By the lower cutoff voltage of 1.08 V, the thickness reduces to a stable value of 129 nm. During charging, the thickness behavior reverses, with the film returning to a stable final thickness of 132 nm at the upper cutoff voltage. Our observations are consistent with previous *in situ* X-ray diffraction experiments.^42,43^

These observations can be contextualized by considering the phase transitions during the lithiation of TiO_2_-anatase. Initially, in Region A (∼1.9–1.6 V), the tetragonal phase (α-TiO_2_: *a* = 3.792 Å, *c* = 9.497 Å, *V*_α_ = 136.56 Å^3^)^38^ undergoes a decrease in *c* and an increase in *a* lattice parameters during lithiation.^42^ In region B, the nucleation and growth of orthorhombic phase (β-TiO_2_: *a* = 3.819 Å, *b* = 4.084 Å, *c* = 9.066 Å, V_β_ = 141.40 Å^3^),^38^ which results in the *a* and *b* lattice parameters becoming different by ∼ 7% and a lattice contraction in the *c* direction by ∼5%.^44^ The gradual depletion of the α-TiO_2_ causes an increase in the film thickness of 2.3% across the Region B plateau, consistent with the expected volume expansion of ∼3.5%. We stress that this thickness measurement is a 1-dimensional measurement and not strictly tracking volume expansion, i.e., no information is obtained on in-plane expansion/contraction. In region C, a small proportion of the high-lithiation-state tetragonal LiTiO_2_ forms (γ-TiO_2_, *a* = 4.043 Å, *c* = 8.628 Å, *V*_α_ = 141.03 Å^3^),^38^ causing a small reduction in the film thickness.

We note that thickness oscillations are observed at the transitions between charge storage regions: ∼1.9 V (Region A), ∼1.6 V (Region A ↔ Region B), and ∼1.4 V (Region B ↔ Region C). Such oscillations were previously seen in thickness monitoring of LiMn_2‑*x*_Ni_*x*_O_4_ thin films by *operando* ellipsometry.^12^ The phenomenon was attributed to how using a single-layer modeling approach handles phase changes, which assumes isotropic phase-change behavior.^12^ This is clearly not the case for TiO_2_-anatase, which undergoes two anisotropic phase changes during lithiation: tetragonal ↔ orthorhombic (α-TiO_2_ ↔ β-TiO_2_) and orthorhombic ↔ tetragonal (β-TiO_2_ ↔ γ-TiO_2_), at the aforementioned voltage regions of ∼1.6 V and ∼1.4 V, respectively, and anisotropic changes during Region A charge storage at ∼1.9 V. Consequently, we propose that it is only appropriate to consider the thickness at the end of each respective Region (A, B and C), in order to avoid overinterpreting thickness changes that might arise due to the fitting process.

### Tracking State of Charge via the Complex Dielectric Constant

Previous ellipsometry studies on the spinels LiMn_2_O_4_ and Li_4_Ti_5_O_12_ demonstrated that the imaginary component of the dielectric function (i.e., relative permittivity) at an appropriate photon energy can be used to track charge storage in these materials.^11,12^ The complex dielectric function is directly related to the complex refractive index: ε­(λ) = *ñ*(λ)^2^ = ε_*r*_(λ) + *i*ε_*i*_(λ) = (*n* ^2^–*k* ^2^) + *i*(2*nk*), where ε*_r_* (λ) and ε*_i_* (λ) represent the real and imaginary components, respectively. It informs about the absorption characteristics of the material, which are highly dependent on the electronic transitions in the optical frequency range (Supporting Figure S8). Therefore, peaks in the ε*_i_* (λ) spectra occur at characteristic wavelengths/photon energies, which in principle can be used to track charge storage.^11^ We repeat the ε*_i_* approach and propose that the normalized ε*_i_* at 1.3 eV can be used to selectively probe the SOC for TiO_2_-anatase films (Supporting Figure S9). In the normalized ε*_i_* vs voltage plot, three distinct regions are observed: region A, B, and C (Supporting Figure S9a, labeled), which correspond to the three TiO_2_-anatase charge storage regions discussed earlier.

We stress that the choice of photon energy to track charge storage is crucial. Specifically, the TiO_2_-anatase system that we study is significantly more optically complex than the previous LiMn_2_O_4_ and Li_4_Ti_5_O_12_ spinel systems reported. The prior spinel materials exhibit 1 or 2 peaks in their ε*_i_* spectra (corresponding to O^2–^ (2p) → TM­[Ni^2+,3+,4+^ or Mn^3+,4+^] (3d) transitions) that increase/decrease in intensity with a change in lithiation state.^12,45^ However, the anatase TiO_2_ system consists of four TiO_2_/Li_*x*_TiO_2_-related ε*_i_* peaks that exhibit red and blue shift, making the choice of photon energy to monitor charge storage non-trivial. This optical complexity arises due to TiO_2_ anatase undergoing a phase change during lithiation, plus the ε*_i_* spectra being dominated by intraband transitions. This is further complicated by the fact that ε*_i_* (λ) does not directly probe the electronic structure of the material. Rather, it monitors the evolution of absorption, which has a strong dependence on the electronic structure but has peak positions that shift as the electronic structure changes during lithiation. Such peak shift has also been reported in several other studies based on reflectivity measurements.^46–48^ Thus, a poor choice of photon energy results in loss of information about charge storage. This issue also persists for optical conductivity (σ_optical_) measurements (Supporting Figure S10), which has also been used to study proton intercalation,^45^ and the absorption coefficient (α) (Supporting Figure S11), due to their proportionality to ε*_i_*. Thus, this highlights the need for a new analytical approach to address the intrinsic peak shift limitation.

### Optical Tracking of Charge Storage via the Energy Loss Function

An alternative approach to probe the electronic structure of a material is energy loss function (ELF). The ELF details how an electron loses energy due to inelastic scattering or a photon is absorbed due to a photoexcitation event, as it passes through a material. Thus, the ELF may be used to map out the density of states of the conduction band. The bulk ELF (the probability of an electron to undergo bulk excitation in volume region) is related to the complex dielectric constant by^49^

ELFbulk=−Im(1ε(E))=εi(E)εr(E)2+εi(E)2 3

where $E\left(\mathrm{eV}\right)=\frac{\mathrm{hc}}{\lambda }$ . In the ELF spectra, peaks at characteristic photon energies/wavelengths correspond to electron excitations, which, when probed in an *operando* manner during an electrochemical process, can be used to track charge storage dynamics in the material. Typically, the ELF is probed with high-energy electrons using electron energy loss spectroscopy (EELS) to probe the core-loss region (energy > 50 eV) via inelastic scattering events. Yet, probing the low-loss region (energy < 50 eV) can provide a wealth of information about interband and intraband transitions, plus the band gap. In electron microscopy, this technique is known as valence EELS (VEELS). But the low-loss region is also accessible to optical techniques using UV–visible–near-IR light sources such as vacuum ultraviolet (VUV) spectroscopy and spectroscopic ellipsometry by monitoring absorption due to photoexcitations by probing the dielectric function.^50,51^ Furthermore, the values of ELF, ε_*r*_(*E*), and ε_*i*_(*E*) are related through the Kramers–Kronig transformations, which have been proven to show optically consistent features in the low-loss region around the band gap of binary and complex oxides (<10 eV).^52^

From the *operando* ellipsometry data collected during CV, the ELF of a TiO_2_-anatase thin film during lithiation is elucidated (Figure 3). The ELF heatmap (Figure 3a) reveals a pronounced peak around 1.3–2.3 eV (Figure 3a, purple dashed rectangle) and a weaker feature in the 2.5–3.4 eV range, which broadens toward the lower cutoff voltage (Figure 3a, indicated by the arrow). These two highlighted changes are indicative of significant changes in the electronic structure, as expected for the optical density spectra of reduced TiO_2_-anatase (transition from Ti^4+^ to Ti^3+^).

**Figure 3:**
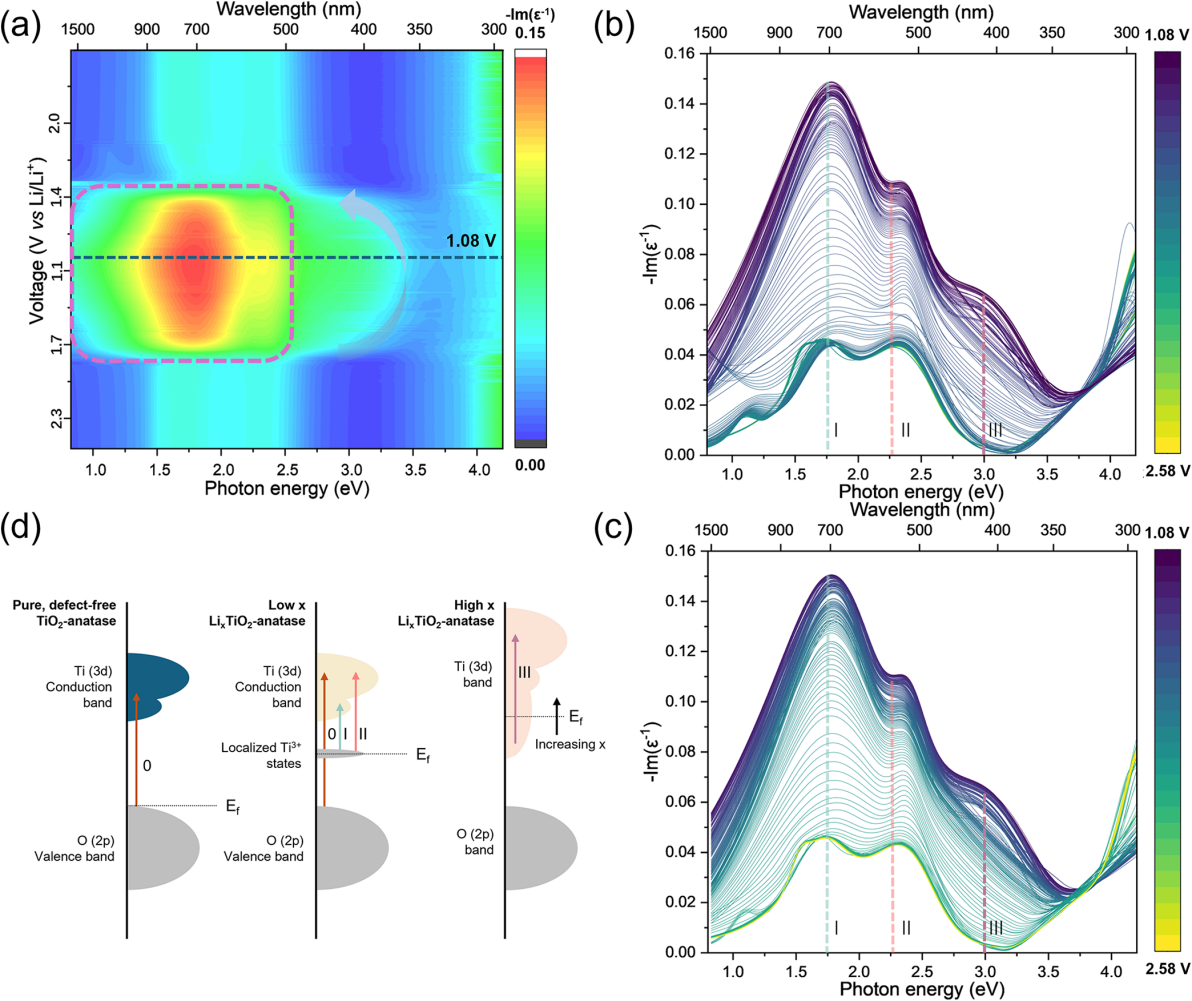
Evolution of ELF spectra of TiO_2_-anatase thin films during CV at 0.1 mV s^–1^, demonstrating a clear reversible change in electronic structure. (a) Heatmap of the ELF during the cycle. The purple dashed box and gray arrow indicate regions where there is a significant change in the ELF spectra. (b, c) Evolution of the ELF 2D spectra during (b) discharging (lithiation) and (c) charging (delithiation). Three clear peaks are present (I, II, III), labeled. A fourth peak (0) above 4.0 eV is observed but not fully resolved in this photon energy range. (d) Band structure schematic of the TiO_2_ and LiTiO_2_ phases, explaining the origin of peaks in the ELF spectra.

Closer examination of the 2D spectra reveals two main peaks at around 1.75 eV (710 nm) and 2.3 eV (540 nm) (Figure 3b,c, Peaks I, II), accompanied by a change in the peak intensity around 4 eV (Figure 3b,c, Peak 0). At lower voltages (V < ∼ 1.6 V), a new peak around 3 eV forms (Figure 3b,c, Peak III), associated with a high degree of lithiation. The band gap evolution was determined by analyzing the change in band gap (*E*_g_ parameter) of the Tauc-Lorentz component of the fitted complex dielectric function. The direct and indirect band gap values are further investigated by traditional Tauc-plot analysis of the absorption coefficient (Supporting Figures S12 and S13). For TiO_2_ at 2.58 V, the indirect band gap is measured as 3.15 eV (Supporting Figure S12a), while the direct band gap is estimated as 3.9 eV (Supporting Information, Figure S12b). Upon lithiation (Li_*x*_TiO_2_) at 1.08 V, the indirect band gap increases to ∼3.5 eV (Supporting Figure S12a), and it is projected that the direct band gap decreases (Supporting Information, Figure S12b); however, full determination of the band edge is limited by the photon energy range of the measurement. This apparent indirect band gap increase is ascribed to occupation of the lowest energy states in the conduction band resulting in Moss–Burstein shift.^53,54^

Assignment of these peaks can be understood by considering the band structures of TiO_2_-anatase and Li_*x*_TiO_2_-anatase (Figure 3d). In TiO_2_-anatase, the valence band has mainly O (2p) character and the conduction band consists mainly of Ti (3d) states, with an indirect band gap of ∼3.2 eV.^55–57^ In TiO_2_ anatase, the Ti (3d) band is reported to split due to long-range band/crystal structure effect,^55–57^ with the Fermi level (*E*_F_) sitting in the band gap just above the valence band.^57^ Peak 0, onset above 3.2 eV, corresponds to the valence band O (2p) → conduction band Ti^4+^ (3d) transition in TiO_2_-anatase, which is not fully resolved in this photon energy range. Upon the formation of Ti^3+^ states during lithiation (Li_*x*_TiO_2_), this peak decreases in intensity due to the depletion of Ti^4+^ states. During lithiation to Li_*x*_TiO_2_, the Li (2s) lies well above the Ti (3d) levels that make up the conduction band.^54^ Subsequently, the Li (2s) electrons enter the lowest unoccupied energy levels located in the Ti (3d) conduction band, accompanied by *E*_F_ rising to sit in the Ti (3d) band.^57^ Peak I and II are intraband Ti (3d) transitions in Ti^3+^ with photon energies smaller than the band gap of TiO_2_-anatase. Two peaks are present corresponding to the split Ti­(3d) conduction band, which arise due to the splitting of the t_2_g and e_g_ orbitals, consistent with previous band structure modeling, EELS, and XAS studies.^56–59^ Note, however, these peaks do not reduce to zero intensity at the upper cutoff voltage (delithiated state), which might be anticipated for the TiO_2_ (Ti^4+^) state. This is because oxygen vacancies result in localized electronic states with Ti^3+^ species character that sit within the band gap.^55^ These are likely induced by either the TiO_2_-anatase/Au interface^60^ or surface oxygen substoichiometry.^55^ Nonetheless, their presence does not affect the ability to track charge storage. During lithiation (discharge to a lower cutoff voltage of 1.08 V vs Li/Li^+^), the intensities of Peaks I and II increase due to the formation of the Ti^3+^ states in Li_*x*_TiO_2_. This is accompanied by *E*_F_ rising to sit in the Ti (3d) band.^57^ Peak III is also an intraband Ti (3d) transition associated with the high-lithiation-state Li_*x*_TiO_2_.^57^ It is only pronounced at low voltages vs Li/Li^+^ when high lithiation levels in the Li_*x*_TiO_2_ film are present. In Li_1_TiO2, the 3d conduction band increases in width, with a new energy band that sits higher than the split Ti (3d) of TiO_2_ anatase, which becomes accessible due to significant electron population in the Ti (3d) band.^57^

### Choosing an Appropriate Wavelength

From a visual inspection of the ELF spectra in Figure 3b,c, clearly there are varying degrees of change in the spectral intensities for a given photon energy/wavelength. Therefore, it is imperative to understand which photon energy tracks all redox processes (Regions A, B, and C) accurately. This is in order to determine when current contributions arise from Ti^3+^/Ti^4+^ redox processes in the TiO_2_-anatase thin film or other charge storage processes. By investigating the evolution of the ELF, we identify Peak II (at 2.3 eV) as the optimal choice to track Faradaic charge storage in our TiO_2_-anatase thin films, which we discuss below.

A comparison of the evolution of the ELF at four distinct photon energies is presented in Figure 4: 1.75 eV (Peak I), 2.3 eV (Peak II), 3 eV (Peak III), and 3.75 eV. First, we consider the evolution of Peaks I, II, and III. Overall, the change in intensity of Peak I (Δ-Im­(ε^–1^) ≈ 0.1) is larger than Peak II (Δ-Im­(ε^–1^) ≈ 0.08) and Peak III (Δ-Im­(ε^–1^) ≈ 0.06). However, this does not directly correlate with sensitivity to Region A, B, and C charge storage.

**Figure 4:**
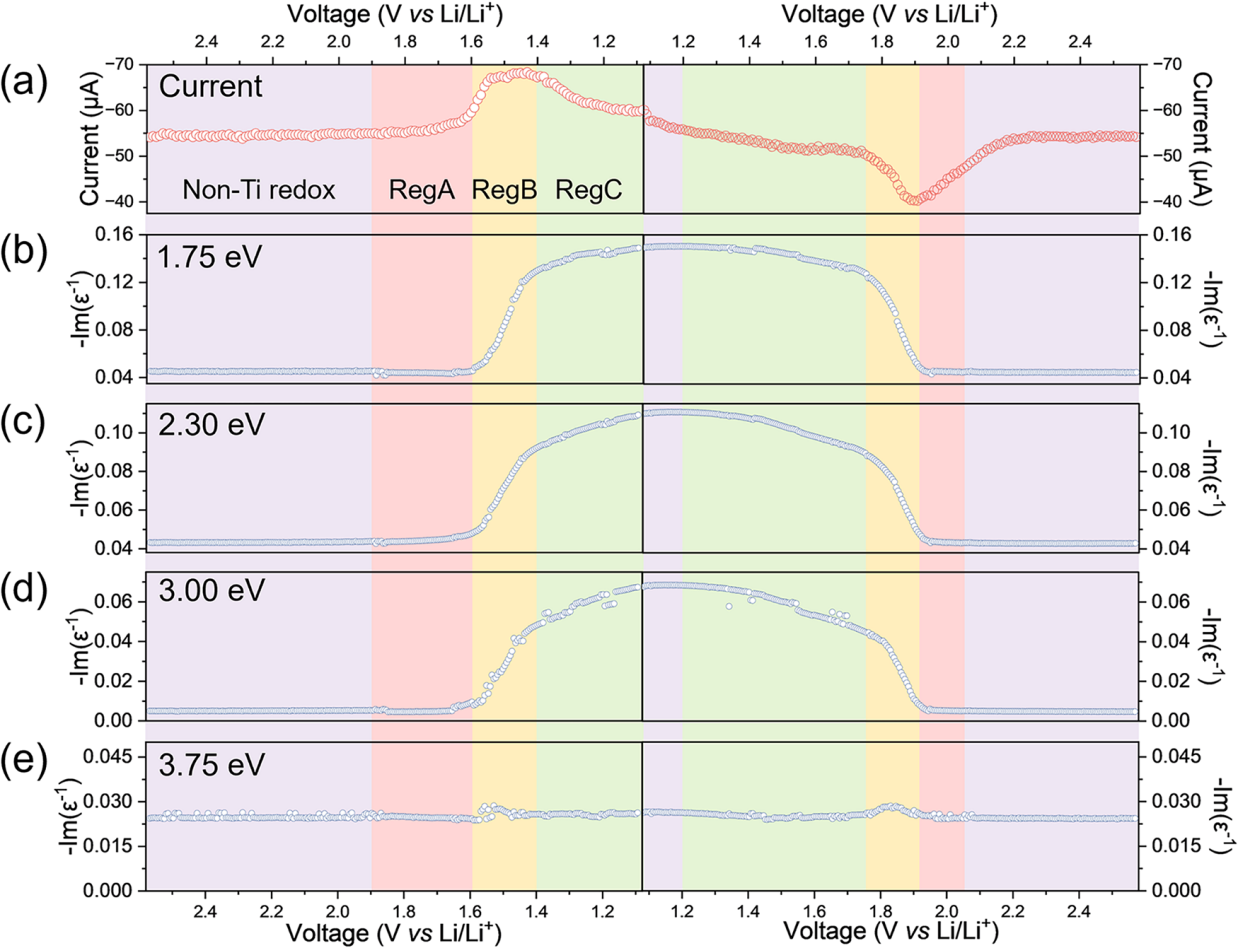
ELF (-Im­(ε-1)) peak analysis to choose a suitable photon energy to track charge storage during CV at 0.1 mV s^‑1^. (a) Cyclic voltammogram current response; evolution of the ELF at (b) 1.75 eV, (c) 2.3 eV, (d) 3.0 eV, and (e) 3.75 eV. The colored shaded regions correspond to the three charge storage regions in TiO_2_-anatase: A (red), B (yellow), and C (green). Areas where there is no change in the ELF response and a nonzero current are assigned to non-Ti redox processes (purple). We find that (c) 2.30 eV has the best sensitivity to the TiO2-redox processes.

Initially, during discharge between 2.58 and 1.9 V vs Li/Li^+^, there is a stable background current in the CV (ca. −55 μA) and no change in the ELF function. Peaks I, II, and III exhibit significant changes in their ELF spectra vs voltage between ∼1.6 and ∼1.4 V during discharge and ∼1.75 and ∼1.90 V during charging. These changes correspond to Region B charge storage in TiO_2_-anatase. Additionally, all three Peaks (I, II, III) observe changes in their ELF spectra between 1.4 and 1.08 V (discharging) and between 1.2 and ∼1.75 V (charging), assigned to Region C charge storage. We note that Region B and Region C charge storage are detected in the same potential range irrespective of the ELF peak (I, II, III) chosen. A deviation in the ELF spectra for all three Peaks (I, II, III) between 1.9 and 1.6 V during discharge and between 1.9 and 2.05 V during charging is observed (Figure 4, red), assigned to Region A charge storage. Here, Peak II exhibits the largest change in the ELF and, thus, the highest sensitivity to Region A charge storage. During charging, there are two regions where there are notable changes in current without a change in the ELF spectra for either Peak (I, II or III) (Figure 4, purple): 1.08–1.2 V and 2.05–2.58 V. As there is no change in the ELF spectra (thus no change in the titanium oxidation state due to delithiation), it can be deduced there is no Ti redox contribution to current in these regions. These two regions are therefore considered to be dominated by other non-Ti redox processes. By the end of the full cycle, the background current of the CV returns to a stable value (ca. −55 μA), and the three ELF spectra return to their original values, indicating a highly reversible TiO_2_-anatase charge storage.

We also track the charge storage at 3.75 eV. Physically, this photon energy does not correspond to an optical transition. From monitoring the evolution of the ELF (Figure 4d), there is clearly minimal change in the spectra, with only 2 small peaks in the Region B area observed. Thus, nothing can be deduced about charge storage at this photon energy. It should be stressed that this does not mean there is no electrochemistry occurring in the sample (as proven by tracking Peaks I, II, and III). While this is an exaggerated example, it demonstrates the need to choose a suitable photon energy to track charge storage with the ELF; if a poor choice is made, limited/no information can be deduced. Overall, we identify that 2.3 eV (Peak II) is the most appropriate photon energy to track charge storage in our TiO_2_-anatase film via the ELF. While Peak I and III adequately track charge storage, Peak II has the highest sensitivity to Region A charge storage. Also, there is a small discontinuity in Peak III ELF data during Region C charge storage, which can be mitigated by choosing a different photon energy.

### Opto-Charge-Discharge Curves and Opto-Voltammograms

We propose the normalized ELF and d­(ELF)/dV at 2.3 eV as a tool to selectively probe the SOC and kinetics of the Ti^3+^/Ti^4+^ redox reaction in the TiO_2_-anatase thin film electrode, confirming Faradaic charge storage mechanisms within the electrode (Figure 5). Here, the normalized ELF is an opto-charge-discharge curve, and the d­(ELF)/dV plot is an opto-voltammogram,^5^ which is an analogue to a CV or differential capacity analysis that is determined directly from the electrochromic response of the material while under electrochemical control. It is determined from the optical response and thus is tracked independently of the current response of the system. We note that the current response contains a convolution of all electrochemical processes that are occurring at a given voltage (e.g., Li^+^ intercalation process, capacitive contributions such as any double-layer formation, electrolyte side reactions, etc., also accounting for overpotentials). Crucially, the d­(ELF)/dV “opto-voltammogram” approach is selective to both the film and specific redox processes occurring by appropriately tuning to the photon energy (eV) corresponding to a specific optical transition.

**Figure 5:**
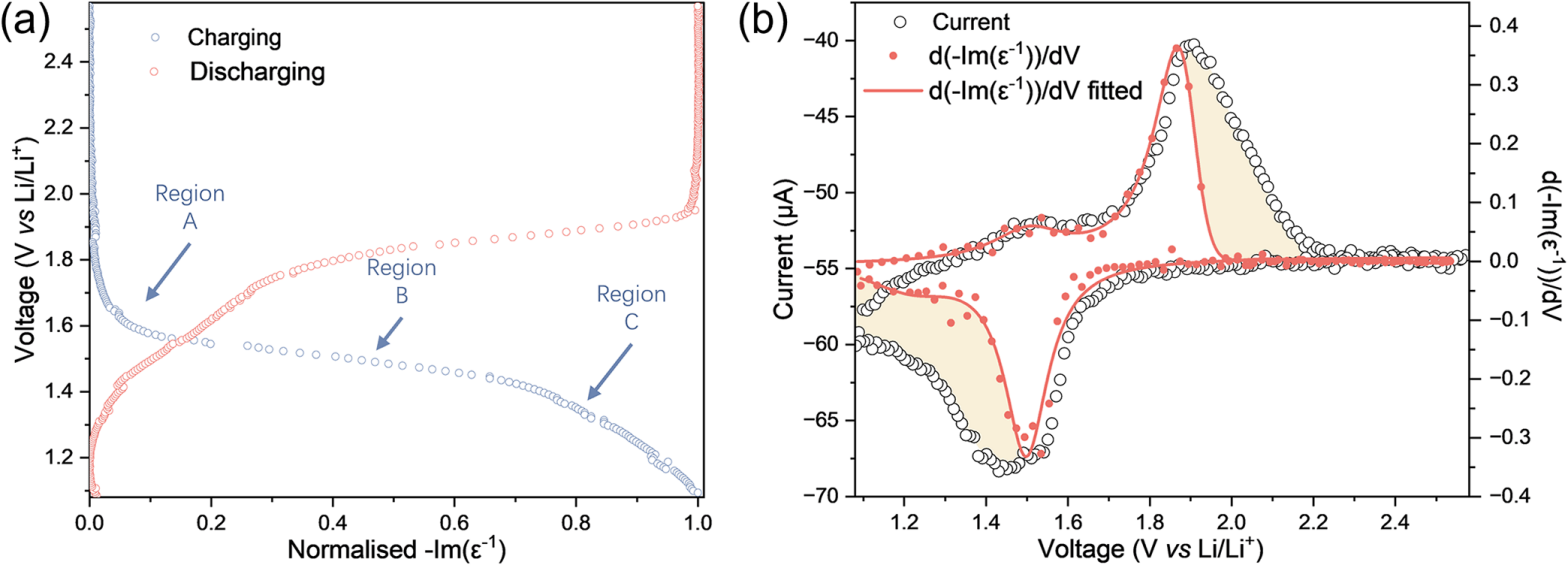
Opto-charge-discharge and opto-voltammograms monitoring the evolution of ELF Peak I at 2.3 eV during CV at 0.1 mV s^–1^. (a) Normalized opto-charge-discharge curve at a photon energy of 2.3 eV. The three charge storage regions of TiO_2_-anatase are clearly resolved (labeled): A, B, C. (b) Opto-voltammogram compared with current response of CV. The yellow-shaded areas indicate regions where the opto-voltammogram and CV deviate away from each other. When d­(-Im­(ε^–1^))/dV = 0 and the current is nonzero, non-Ti redox processes are occurring.

The normalized ELF enables the SOC of the TiO_2_-anatase film to be tracked in an analogous way to a charge-discharge curve (Figure 5a).^11^ Three distinct regions, the aforementioned regions A, B, and C, are observed (labeled in Figure 5a), consistent with previous electrochemical studies.^35–38^ Note here that, unlike the aforementioned electrochemistry papers,^35–38^ we deduce the above information directly from the electrochromic response of the material (ELF). This gives an alternative insight into the charge storage of our TiO_2_-anatase film electrode, as it deconvolutes the contribution from the residual current observed in the CV (Figure 5b). Thus, the ″opto-voltammogram” technique precisely separates pure Faradaic TiO_2_-anatase redox from other processes.

By comparing the electrochromic response (red, Figure 5b) with the current response (black, Figure 5b), we observe that the features d­(ELF)/dV closely match the CV. During charging, d­(ELF)/dV = 0 at 2 V vs Li/Li^+^, faster than the residual current reaching a stable value at 2.3 V vs Li/Li^+^. In other words, there is no Faradaic current contribution from Ti redox of the TiO_2_-anatase film between 2 and 2.3 V vs Li/Li^+^, but the residual current indicates other processes are occurring (e.g., electric double-layer capacitance, (EDLC), or parasitic currents). Similarly, the yellow-shaded region highlighted in Figure 5b indicates the deviation between the TiO_2_-anatase Faradaic current and the CV current. Notably, an additional pair of redox peaks appears in the d­(ELF)/dV plot (at 1.5 V during delithiation and 1.3 V during lithiation), indicating Region C charge storage.^37,61,62^ These are not so apparent in the current response, where the main peak is broadened, i.e., convoluted by all ongoing redox processes.

### Assessing Kinetic Limitations with *Operando* Ellipsometry

Next, we examine the *operando* ellipsometry data while increasing the CV scan rate between 0.1 and 8 mV s^–1^ (Figures 6 and 7) to check the sensitivity of the ELF response. For all scan rates, the characteristic reversible redox peaks associated with the TiO_2_-anatase Ti^3+^/Ti^4+^ redox couple are present (Figure 7a). Inspecting the 2D ELF spectra at CV scan rates between 0.1 and 8 mV s^–1^ (Figure 6, full series reported in Supporting Figure S14 (discharge) and S15 (charge)), it is clear there are progressively smaller changes in the profile with increasing CV scan rates, which is very apparent when comparing the response at 0.1 mV s^–1^ with 4 mV s^–1^ (Figure 6). Recall that for this profile to change, there must be a change in the titanium oxidation state (Ti^3+^ ↔ Ti^4+^) due to a Faradaic reaction during lithiation. As Ti^3+^ forms, the absorption intensifies at 2.3 eV during lithiation. Hence, directly from these 2D ELF spectra, we can determine that there is reduced Li^+^ insertion at the fastest scan rate conditions, indicating a transition to a diffusion-limited regime. The ELF profile across all scan rates (Supporting Information Figures S14 and S15) displays high reversibility. Notably, the peak in ELF at 2.3 eV remains the most significant change observed across the entire scan rate range (Figure 6).

**Figure 6:**
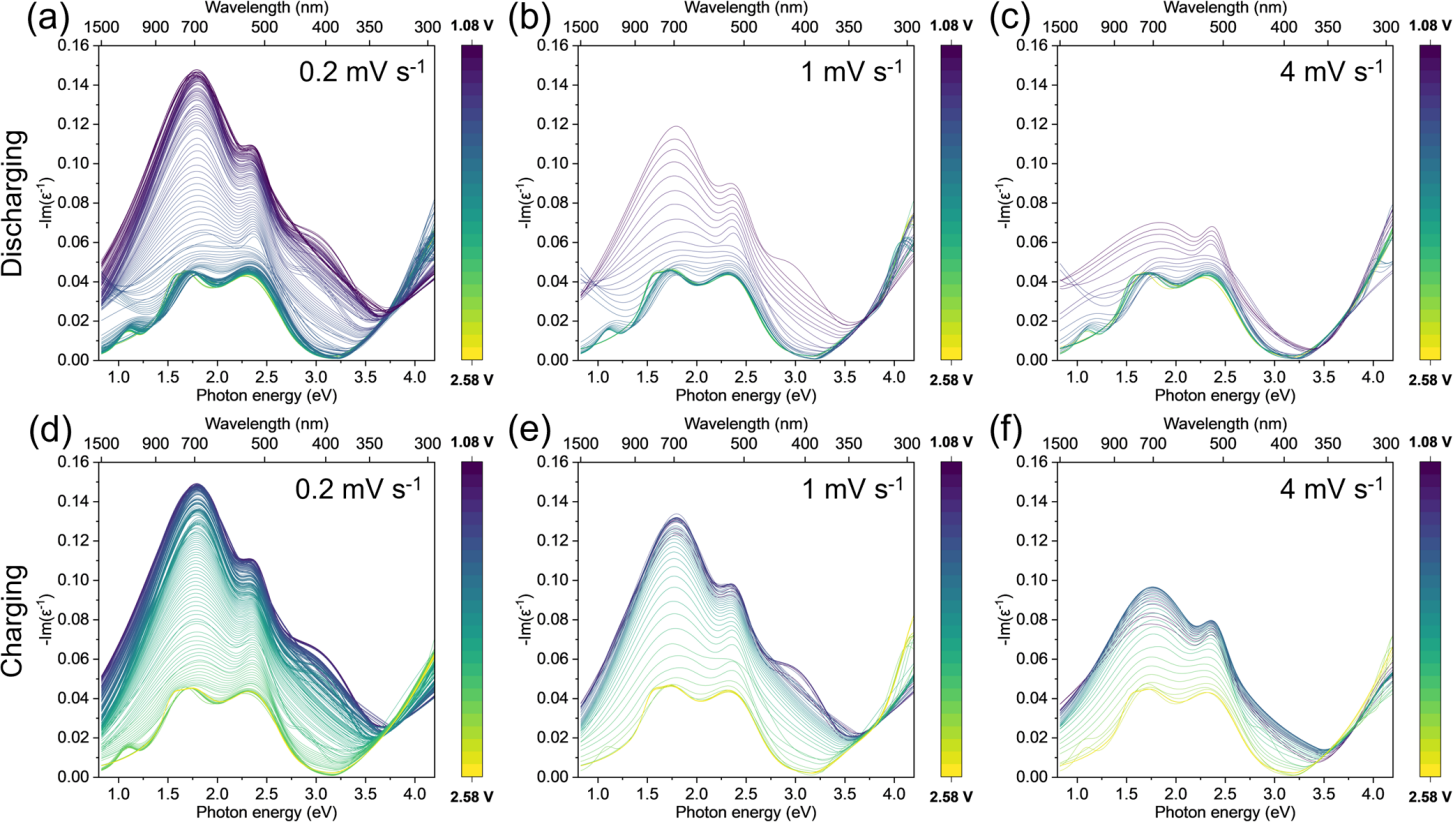
Evolution of selected ELF 2D spectra of TiO_2_-anatase thin films during cycling at increasing scan rates (a–c) during discharge (lithiation) and (d–f) during charging (delithiation) at 0.2, 1, and 4 mV s^–1^, respectively. With increasing scan rate, there is a reduction in the ELF spectral feature intensities indicating kinetic limitations. Also, at 4 mV s^–1^, Peak III, associated with the formation of Li-rich tetragonal Li_0.5+*x*_TiO_2_, does not form, which is also associated with kinetic limitations.

**Figure 7:**
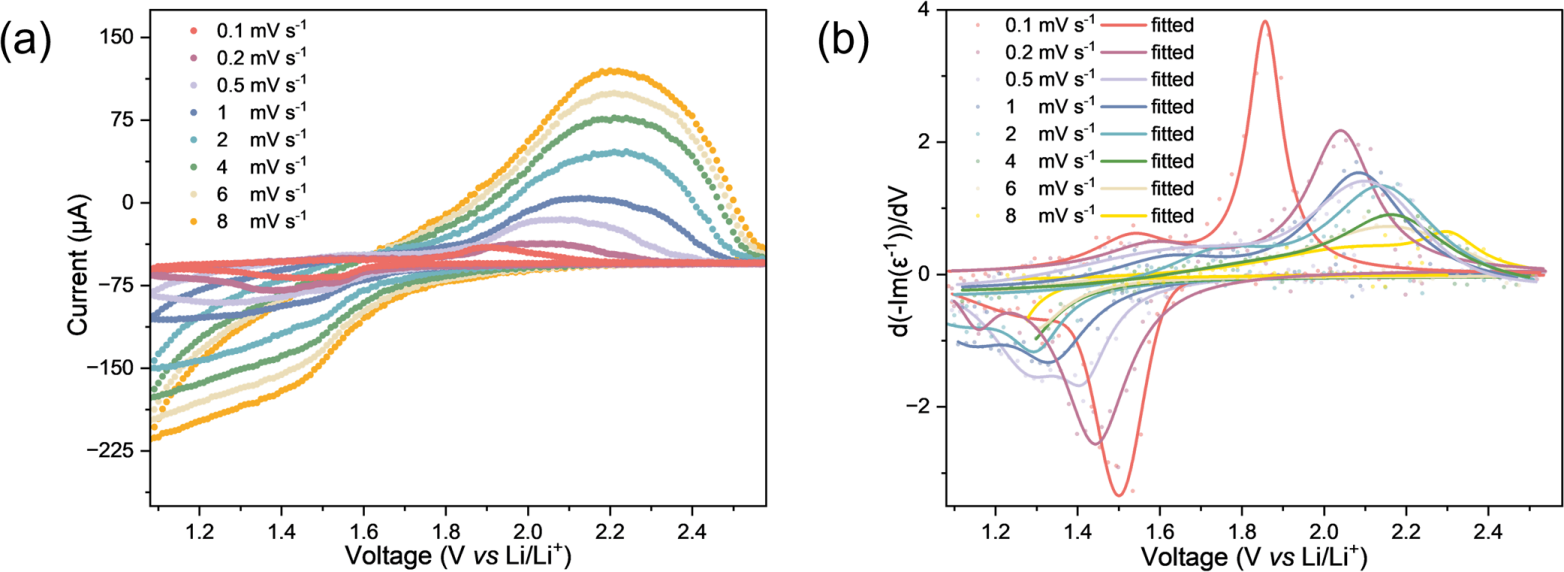
Assessing kinetic limitation with opto-voltammograms. (a) CVs collected at scan rates between 0.1 and 8 mV s^–1^. A single peak is observed, which exhibits signs of overpotential with increasing scan rates. (b) Opto-voltammograms (differential ELF: d­(-Im­(ε^–1^))/dV) for each CV scan rate tested. With increasing scan rate, the peak intensity decreases, indicating reduced Ti redox processes. Likewise, the peak positions shift with increasing scan rate due to overpotential.

With increasing CV scan rate, there is a clear increase in overpotential associated with diffusion limitations under faster scan rate conditions (Figure 7b). Supporting Figure S16 shows the evolution of ELF during discharge with increasing CV scan rate, where we observe a distinct smooth voltage tail (i.e., Region C as defined earlier) existing until 1 mV s^–1^. The overpotential does not affect the onset of the redox reaction (i.e., the ELF starts to change at 1.6 V for all scan rates), while the transition from Region B to Region C charge storage is delayed to more negative voltage with increasing scan rate. At scan rates above 1 mV s^–1^, the Region B plateau in all ELF plots slopes at a greater angle and the Region C plateau is not present. This indicates that the system is operating under diffusion control, limiting the amount of lithiation that can occur in the time frame of the measurement.^63^ The opto-voltammogram of ELF at 2.3 eV at variable scan rates (Figure 7b) displays the dynamic charge response of the electrode. The opto-voltammograms share the same peak positions as the CVs, indicating the same overpotential. Furthermore, a decrease in the d­(ELF)/dV intensity indicates a reduction in charge stored.

To justify the relationship between Faradaic charge storage within the electrode and the ELF, we examine the galvanostatic cycling of the TiO_2_-anatase thin films with *operando* ellipsometry at C-rates of 0.9, 2.2, 3.8, 6.0, and 9.3 C, respectively. We normalize the ELF data to the value at the lower cutoff voltage of 1.08 V vs Li/Li^+^. We observe that the charge-discharge curve (red/blue lines, Figure 8a) closely correlates with the normalized ELF (open markers, Figure 8a; normalized ELF = 1 for max SOC at 0.9 C). Again, three distinct regions are observed, regions A, B, and C (Figure 8a, labeled); these correspond to the three TiO_2_-anatase charge storage regions discussed earlier. These regions are further assigned with a d­(ELF)/dV opto-voltammogram (Figure 8b), which is well correlated with dQ/dV. Notably, compared with CV studies (Figure 5b), Region A charge storage is clearly resolved in galvanostatic cycling during charging (Figure 8b).^35,36^ There is a small deviation in the Region C charge storage of the discharge curve, which arises because the Coulombic efficiency of the cell (93.1%) is less than the optically derived efficiency (>99%) determined from the ELF. This indicates that irreversible capacity loss in the cell is not due to a reduction in charge storage in the TiO_2_ film. The correlation between ELF and the charge-discharge curve also holds when increasing the current density/C-rate, with a reduced maximum in normalized ELF observed at higher rates (Figure 8c). This is consistent with kinetic control limitations under high-rate conditions, resulting in a lowered capacity due to reduced Li^+^ insertion. Peak shift due to overpotential is also observed with increasing current density/C-rate (Figure 8d). Overall, these findings confirm the high sensitivity and selectivity of OE, showcasing its ability to monitor titanium-specific charge storage mechanisms within the electrode.

**Figure 8:**
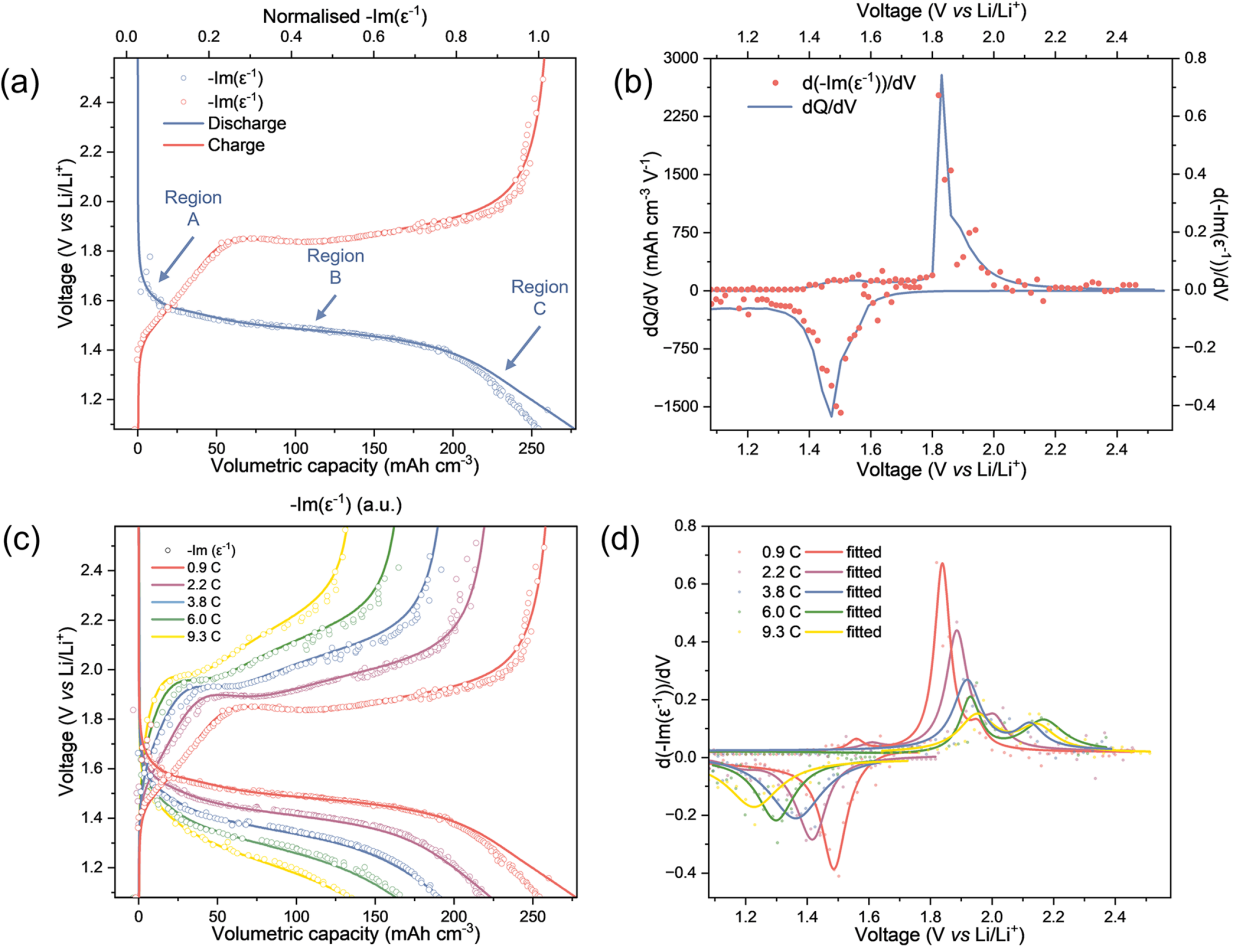
Opto-charge-discharge and opto-voltammogram analysis of TiO_2_-anatase thin film during galvanostatic cycling. (a) Normalized opto-charge-discharge curves (2.3 eV) comparison with volumetric capacity and (b) opto-voltammogram (2.3 eV) and differential capacity (dQ/dV) analysis comparison during cycling at 0.9 C. Close correlation is observed. (c) Rate capacity analysis (0.9–9.3 C) overlaid with opto-voltammogram (2.3 eV) response. Again, a clear correlation is observed. (d) Opto-voltammogram analysis with increasing C-rate. Analogous to CV analysis, peak intensities reduce with increasing C-rate associated with kinetic limitations, and peak shifts are observed associated with overpotential.

## Discussion

This article presents *operando* ellipsometry insights into the charge storage of TiO_2_-anatase thin films. Using the ELF spectra, derived from the complex dielectric function, we demonstrate the ability to track lithiation by monitoring the evolution of a Ti (3d) intraband peak. We build on previous studies that have used the imaginary component of the dielectric function (ε*_i_*) to track charge storage in thin films.^11,12^ While this can be an effective approach for some materials, it can suffer from red/blue-shifted peaks, which complicates assessment. This is particularly prevalent in materials where phase transitions result in significant changes in the band structure, such as the TiO_2_-anatase system studied here (see Supporting Information Figure S12). This complexity arises because ε*_i_* does not directly probe the electronic structure of the material; rather, it monitors the evolution of absorption. While we do find that tracking the evolution of ε*_i_* at 1.3 eV enables tracking of the charge storage in our TiO_2_-anatase film (Supporting Information Figure S9), deconvolution of the Ti redox process might not be guaranteed due to the red/blue shifting of this peak during (de)­lithiation.

On the other hand, this intrinsic limitation can be overcome by analyzing the ELF spectra. Because the ELF probes the electronic structure of the material, its spectral features do not exhibit a red/blue shift. Thus, a characteristic peak/photon energy can be chosen to monitor charge storage, the significance of which can be assigned by considering the band structure of the material. In the case of TiO_2_-anatase, we find that the ELF spectral peak at 2.3 eV (539 nm) corresponding to a Ti (3d) intraband transition provides the greatest insights into charge storage (Figure 4). Thus, the normalized ELF can be used as an opto-charge-discharge curve, and the derivative as an opto-voltammogram, to characterize the charge storage mechanism directly with light during cyclic voltammetry (Figures 5 and 7) or galvanostatic cycling (Figure 8). Specifically, we resolve the three main constituents of charge storage in TiO_2_-anatase: Region A, B, and C charge storage independently of the current response (Figure 5a and Figure 8a). By comparison with the electrochemical data (which probe the charge storage of the whole cell), our *operando* ellipsometry ELF approach can deconvolute Ti redox processes (Figure 5b). Additionally, using our ELF approach, we can monitor the lithiation kinetics in the film over a wide range of CV scan rates/C-rates (Figures 6 and 7).

Finally, a key consideration is the choice of light source to probe the system. In the broader context of the field, probing battery materials with optical photons (either a single-wavelength lasers or white light) to visualize charge storage is approaching widespread commercialization, with techniques such as charge photometry, optical fiber sensing, and reflectometry recently reaching market.^6,64,65^ Yet, utilization of such approaches leaves several pertinent questions: which wavelength region can provide the most relevant information? Does the use of a single wavelength severely limit the information obtainable about the system? In the case of *operando* ellipsometry, we identify that choosing a suitable wavelength/photon energy is crucial to avoid information being lost (Figure 4). This is because certain wavelengths or energies may not be sensitive to the ongoing electrochemical process. In particular, photon energies greater than the band gap (which is material-specific and can span the whole UV–visible–near-IR range) could be severely restricted in probing charge storage (Figure 4e). This is because photons of such energies are strongly absorbed by the film (Supporting Figure S11), thus preventing the whole depth of the electrode from being probed, which is particularly pertinent when monitoring volumetric changes. Thus, extreme care should be taken if an arbitrary wavelength is chosen (either due to spectrometer restrictions or user choice), as it may not be sensitive to the ongoing electrochemical process. In such situations, an advantage of *operando* ellipsometry is the ability to probe a broad spectral range, from UV to near-IR, providing both real (amplitude) and imaginary (phase) information about the reflectance of the thin film structure with sufficiently quick time resolution (<10 s). Thus, ellipsometry is well suited as an experimental method which, when paired with band structure modelling, can provide complementary information about relevant electrode materials.

## Conclusion

To summarize, we demonstrate that *operando* ellipsometry can be utilized to investigate the charge storage mechanisms of lithium-ion battery electrodes on a model TiO_2_-anatase film. First, we demonstrate that monitoring the evolution of a characteristic peak of the energy loss function (ELF at 2.3 eV) enables precise tracking of the lithiation state (state of charge, SOC) in an *operando* manner while under electrochemical control. Thereafter, we introduce the opto-voltammograms d­(ELF)/dV, an optical analogue differential capacity analysis (d*Q*/d*V*), and cyclic voltammetry derived from the electrochromic response of the material. The key advantage of d­(ELF)/dV is that, by choosing an appropriate photon energy, it is selective to the Faradaic redox process occurring in the film. Crucially, this enables critical assessment of kinetic limitations during charge storage, deconvolution from other ongoing electrochemical processes such as interphase formation, electrolyte side reactions, and double-layer capacitance, and thus unambiguous assignment of pseudocapacitive charge storage. Our findings establish OE as a promising tool for a greater understanding of charge storage and surface phenomena in next-generation batteries.

## Methodology

### Preparation of TiO_2_-Anatase Thin Films

The preparation method for the pure TiO_2_ films was adapted from ref 66. TiO_2_ inorganic sol was prepared by adding 0.193 mL of titanium isopropoxide (TiOPr, >98%, Merck) to 0.061 mL of HCl (37%) under constant stirring. The resulting sol was diluted with 0.15 mL of ethanol. The prepared sol was spin-coated onto 2.3 × 2.3 cm^2^ gold-coated silicon wafers (100 nm Au on silicon with 10 nm adhesion layer of TiO_2_, Amsbio) at 2000 rpm for 1 min. The samples were then calcined in a muffle furnace at 450 °C for 30 min.

### Spectroscopic Ellipsometry and *Operando* Ellipsometry

All measurements were made by using a Semilab SE-2000 rotating compensator (RC) spectroscopic ellipsometer. RC ellipsometry measures spectral parameters Ψ and Δ, which correspond respectively to the amplitude ratio and the phase difference of the complex reflection coefficients of light polarized parallel and perpendicular to the plane of incidence. For all ex situ and *operando* measurements, the acquisition time was set to 10 s. The measurements were carried out at an incidence angle of 70° in the wavelength range of 295–1600 nm. The *operando* measurements were performed in a specialized EC-Ellipsometry cell (Redox.me). The optical data were analyzed with Semilab SEA software to extract the thicknesses and complex dielectric function parameters for each measurement point. Optical modeling of the TiO_2_-anatase layer was achieved by utilizing a combination of Tauc-Lorentz and Drude dispersion laws, combined further with two Lorentzian and three Gaussian oscillators (see Supporting Information for more details). A combination of global search, semi-global, and local search algorithms was utilized to ensure that the best overall fit is achieved without getting stuck in local solution. This combination uses six passes with different regression fitting algorithms: first pass Grid search, second pass Levenberg–Marquardt algorithm (LMA), third pass Simplex, fourth pass LMA, fifth pass Price’s algorithm, and sixth pass LMA. Each pass optimizes different parameters of the dispersion laws/oscillators, as detailed in Supporting Table S1. Each measurement data point (in time) was fitted independently with this algorithm to ensure reliable regression (goodness of fit R^2^ > 0.995).

### Electrochemical Characterization

All electrochemical testing of TiO_2_-anatase thin film electrodes was performed using an EC-Ellipsometry Cell (Redox.me). Prior to cell assembly, the samples were cleaned in ethanol and heated at 100 °C in a vacuum oven overnight to eliminate residual moisture. The gold-coated silicon wafers were subsequently integrated into an EC-Ellipsometry cell (Redox.me), where they served as the working electrode against a Pt wire counter electrode and a nonaqueous Ag/Ag^+^ reference electrode (0.01 M AgNO_3_ and 0.1 M TBAP in acetonitrile, Redox.me). The electrolyte used was 3 mL of 1 M LiClO_4_ in propylene carbonate (PC). All voltages are reported vs Li/Li^+^, converted from the nonaqueous Ag/Ag^+^ reference using the relation: 0 V­(Ag/Ag^+^) = 3.08 V­(Li/Li^+^). All assembly steps were carried out in an Ar-filled MBRAUN glovebox (O_2_ < 1 ppm, H_2_O < 1 ppm). All CV experiments were performed at scanning rates of 0.1, 0.2, 0.5, 1, 2, 4, 6, and 8 mV s^–1^ between 1.08 and 2.58 V vs Li/Li^+^. The cycling performance of the materials was studied by galvanostatic cycling between 1.08 and 2.58 V vs Li/Li^+^ at current rates of 0.9, 2.2, 3.8, 6, and 9.3 C (where 1 C corresponds to 3.5 μA cm^–2^, the calculations of C-rates were based on the maximum volumetric capacity of 275 mAh cm^–3^ for 1 h discharge).

### Atomic Force Microscopy

Atomic force microscopy (AFM) experiments were conducted using a Bruker Dimensions Icon with ScanAsyst housed in an Ar-filled glovebox (<0.5 ppm of H_2_O and O_2_), equipped with Bruker RTESPA-300 AFM tips (Sb­(*n*) doped Si with reflective Al coating, *k* = 40 N m^–1^¸ *f*_0_ = 200 kHz). The topography of the film surface was mapped with the ScanAsyst mode. All AFM images were analyzed using the Gwyddion software.^67^

### Scanning Electron Microscopy

Cross-sectional scanning electron microscopy (SEM) was performed using a Zeiss Crossbeam 540 instrument with a 15 kV accelerating voltage. The sample (TiO_2_ film post *operando* ellipsometry) was cross-sectioned with a diamond scribe to create a cleaved surface.

### Raman Spectroscopy Mapping

Raman mapping was conducted on a film pre- and post-CV using a Renishaw inVia micro-Raman spectrometer equipped with a 785 nm laser and a 2400 lines per mm grating. Surface Raman spectra were captured using a Renishaw CCD Camera with a 20x objective lens, with the 785 nm edge laser (mode: Linefocus) operating at 5% power within a wavenumber range of 100–1000 cm^–1^, with each spectrum accumulated over two 30-s scans. A rectangular map consisting of 121 spectra was generated over a 400 μm-by-400 μm area. Peaks at Raman shifts of 400 cm^–1^, 520 cm^–1^, and 630 cm^–1^ were fitted using a custom Python batch fitting script with multiple Lorentzian functions, while the background was removed using the Baseline Removal Python module, following ref 68. The resulting intensity map of the 520 cm^–1^ peak, showing the background-subtracted maximum vertical height, was generated using an extended Python code and then plotted.

## Supplementary Material



## Notes

Raman mapping of uncycled and cycled TiO_2_-anatase films, AFM topography images, cross-sectional SEM, baseline ellipsometry spectra, selected fitted ellipsometry spectra, details of fitting algorithm, refractive index plus extinction coefficient determined from *operando* ellipsometry, measured film thickness during *operando* ellipsometry, complex dielectric constant, opto-charge-discharge curves determined from ε*_i_*, optical conductivity, adsorption coefficient, Tauc plots, band gap evolution during *operando* ellipsometry, ELF evolution during *operando* ellipsometry, and opto-charge curve (PDF)
