# Supplementary material for: Tracking Lithium Intercalation in Battery Electrodes via Their Electrochromic Properties Using Operando Ellipsometry
Source: J Am Chem Soc. 2025 Oct 21;147(44):40967–80. doi: 10.1021/jacs.5c14539 (PMC12593327; doi:10.1021/jacs.5c14539)
Supplement: Supplementary file 1 [file ja5c14539_si_001.pdf]

Supporting Information: Tracking Lithium Intercalation in Battery Electrodes via Their Electrochromic Properties Using *Operando* Ellipsometry

Jialin Gu[1], Adam J. Lovett[1,2,3*], Máté Füredi[1,4], Thomas Dore[1,2], Stefan Guldin[1,5,6], Thomas S. Miller[1,2,3*]

[1]Department of Chemical Engineering, University College London, Torrington Place, London, United Kingdom, WC1E 7JE

[2]Advanced Propulsion Lab, Marshgate, University College London, London, United Kingdom, E20 2AE

[3]The Faraday Institution, Quad One, Didcot, United Kingdom, OX11 ORA

[4]Semilab Co. Ltd., Prielle Kornélia u. 2, Budapest, Hungary, H-1117

[5]Department of Life Science Engineering, Technical University of Munich, Freising, Germany, 85354

[6]TUMCREATE, 1 CREATE Way, #10-02 CREATE Tower, 138602, Singapore

*Correspondences: adam.lovett@ucl.ac.uk; t.miller@ucl.ac.uk

Table of Contents

Supporting Figures **S2**

1. Additional Structural Characterization Data **S2**

Figure S1: Raman mapping of uncycled and cycled TiO_2-anatase films S2

Figure S2: Atomic force microscopy topography images S3

Figure S3: Cross-section scanning electron microscopy images S3

2. Ellipsometry Modeling S4

Figure S4: Baseline ellipsometry spectra S4

Figure S5: Example fitted *operando* ellipsometry data S5

Table S1: Details of ellipsometry fitting algorithms S6

3. Parameters Extracted from *Operando* Ellipsometry S7

Figure S6: Evolution of refractive index and extinction coefficient S7

Figure S7: Measured film thickness during *operando* ellipsometry S7

Figure S8: Complex dielectric constant (ε_r, ε_i) evolution during cycling S8

Figure S9: Opto-charge-discharge curve & opto-voltammogram from ε_i S9

Figure S10: Optical conductivity during *operando* ellipsometry S9

Figure S11: Adsorption coefficient during *operando* ellipsometry S10

Figure S12: Tauc plots to determine band gap S10

Figure S13: Evolution of band gap during *operando* ellipsometry S11

Figure S14: ELF discharging evolution S11

Figure S15: ELF charging evolution S12

Figure S16: Opto-charge curve S12

Supporting References S13

Supporting Figures

1. Additional Structural Characterization Data

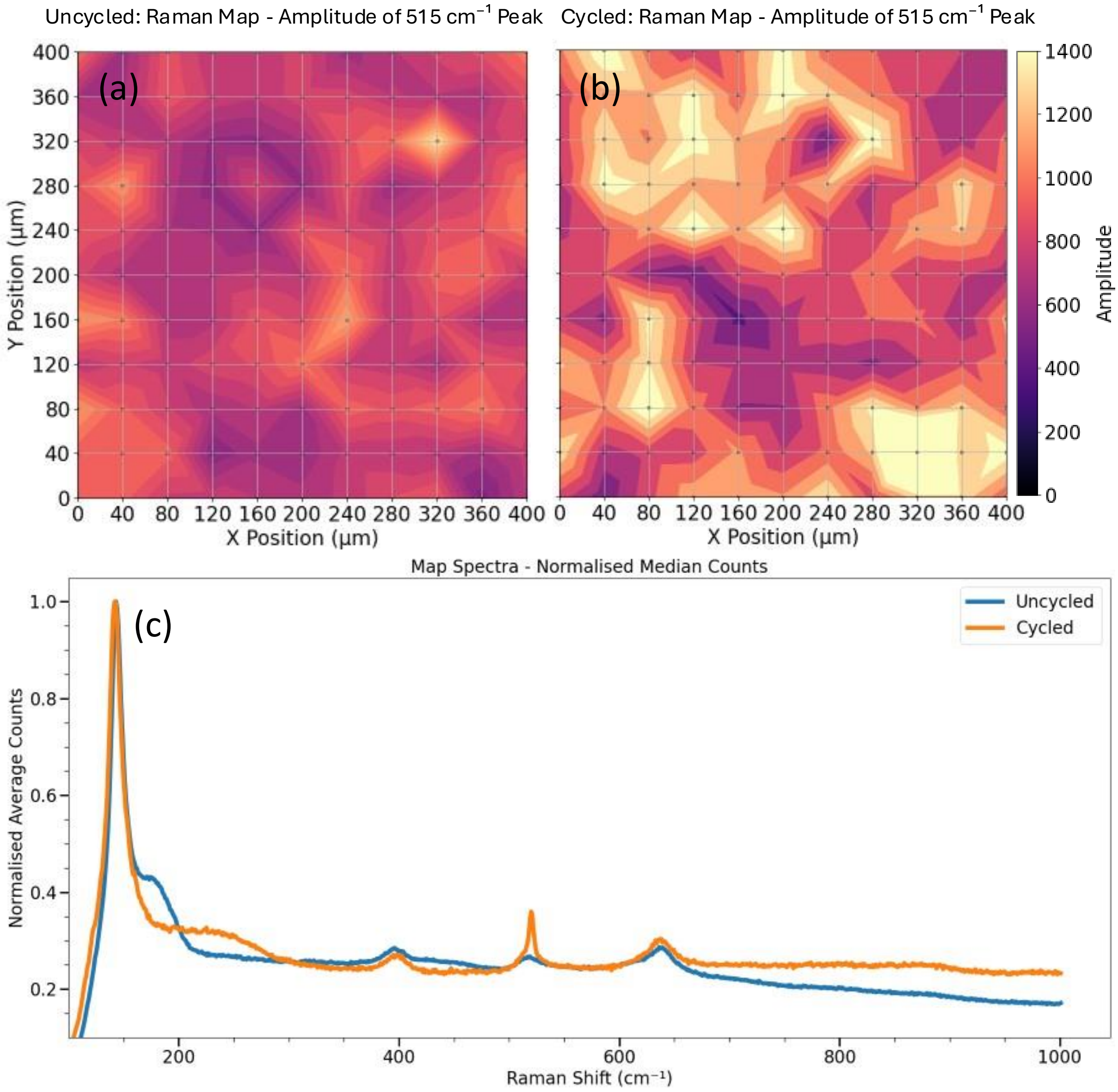

Figure S1: Figure S3: Raman mapping of TiO_2-anatase films, illustrating the spatial distribution of characteristic Raman peaks of the anatase phase. (a) Raman map of the amplitude of the characteristic anatase peak at 515 cm^{-1} for the uncycled sample, obtained from 121-point spectra across a 400 µm × 400 µm area. (b) Corresponding Raman map for the cycled sample, also based on 121-point spectra over a 400 µm × 400 µm area. (c) Averaged Raman spectra for both the uncycled (A) and cycled (B) maps, normalised to the amplitude of the 143 cm^{-1} peak. To evaluate a representative Raman spectrum across the whole region of each map, the median Raman spectrum comprised of all 121-points was calculated. The median plot was chosen so that cosmic rays do not bias the counts for a given Raman shift. The broad peak at 230 cm^{-1} in the cycled spectra (Orange) is ascribed to lithiated TiO_2-anatase [1,2].

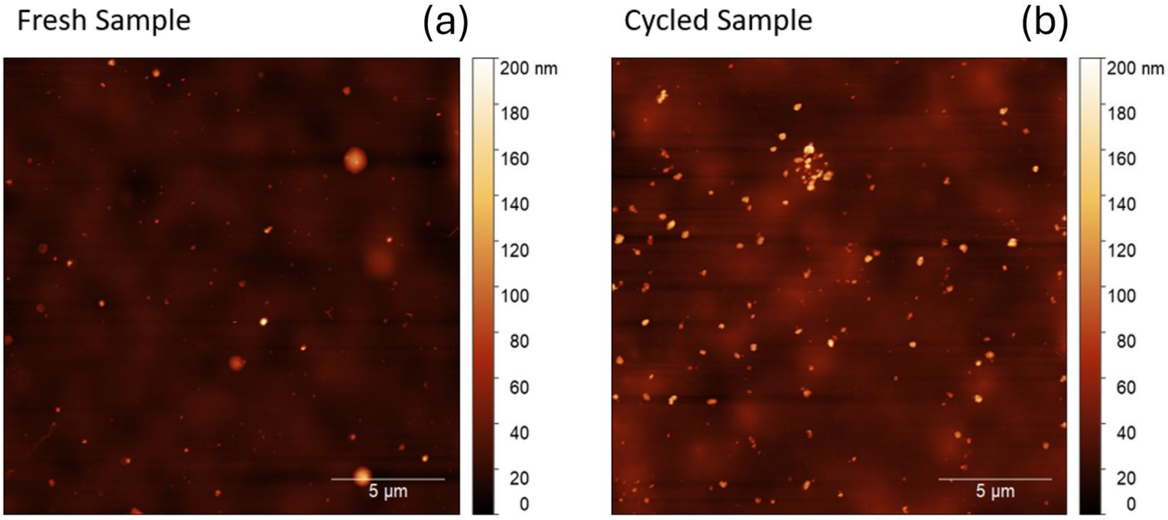

Figure S2: Atomic Force Microscopy (AFM) morphology of TiO_2-anatase films. (a) TiO_2-anatase film before cycling. (b) TiO_2-anatase film after cycling.

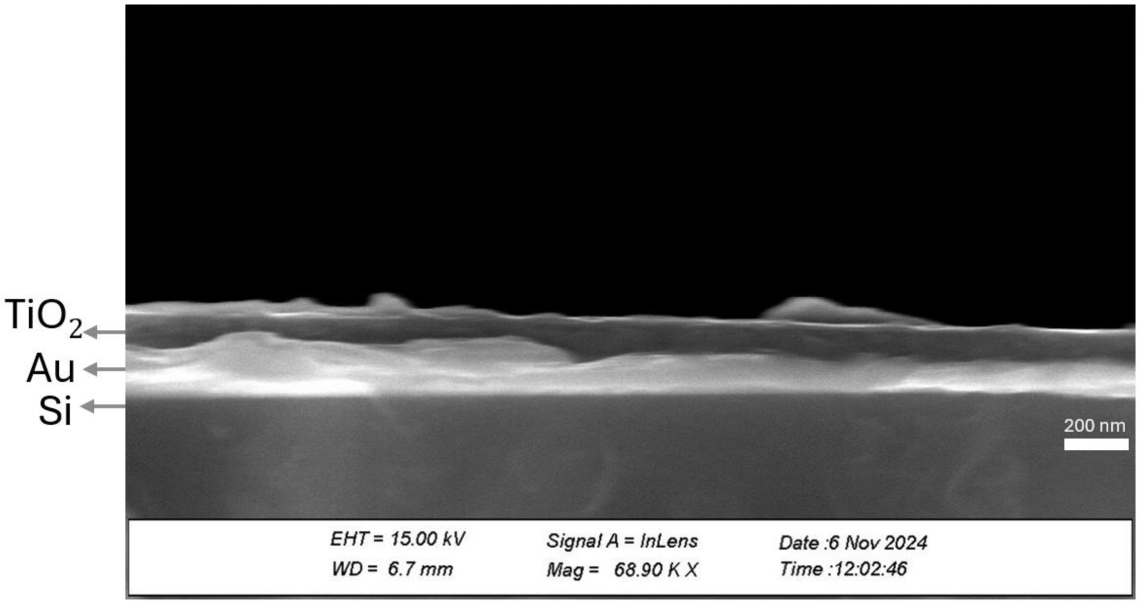

Figure S3: Cross-sectional scanning electron microscopy (SEM) image of TiO_2-anatase films post *operando* ellipsometry.

2. Ellipsometry Modelling

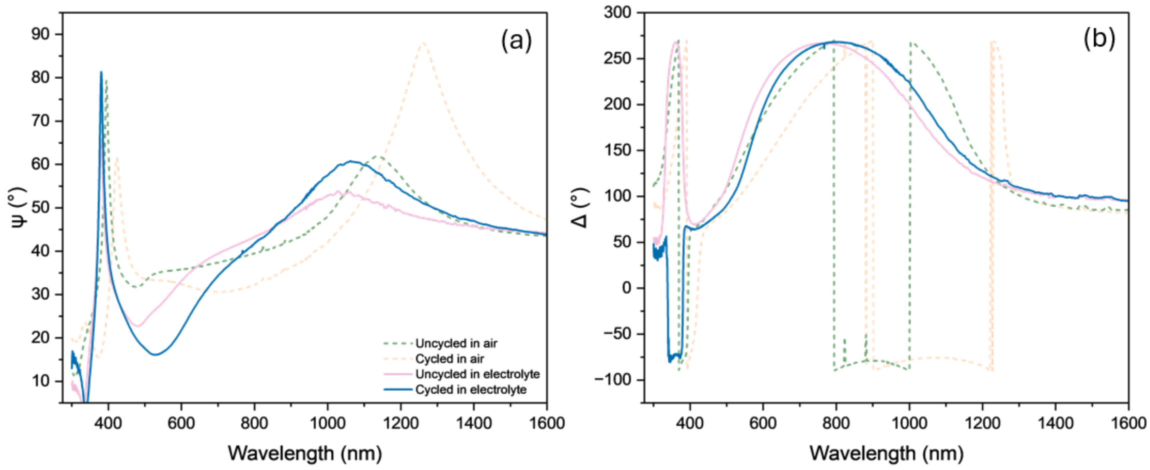

Figure S4: Baseline ellipsometry spectra of the TiO_2-anatase films. (a) Amplitude ratio (Ψ) and (b) phase difference (Δ). The baseline ellipsometry measurements in air (green dashed) are compared with *in situ* ellipsometry collected at open current voltage in electrolyte (pink), post cyclic voltammetry cycling (blue) and *ex situ* in air post CV (dashed yellow). Slight shifts in the spectra are observed. The differences between in air and in electrolyte ellipsometric spectra occurs due to measurement medium having different optical properties (air n = 1, PC n = 1.4). The cycled samples shift due to structural changes that occur during cycling, such as lithiation, film thickness changes or solid-electrolyte interphase contributions.

The mathematical description of the optical modelling is described below with Tauc-Lorentz and Drude dispersion laws, combined further with Lorentzian and Gaussian oscillators (j, and k number of oscillators respectively). The model is generally applicable to oxide semiconductors with intraband transitions and varying lithiation states.

If $E > E_g$:

$$\varepsilon_i(E) = \frac{1}{E}\frac{AE_0C\left(E - E_g\right)^2}{\left(E^2 - {E_0}^2\right)^2 + C^2E^2} + \frac{{E_p}^2E_\Gamma}{E(E^2 + {E_\Gamma}^2)} + \sum_j\left(\frac{f_j(E\Gamma_j)}{\left((E_j^{center})^2 - E^2\right)^2 + \left(E\Gamma_j\right)^2}\right) + \sum_k\left(B_k\, exp\left(\frac{(E - E_k^G)^2}{2{\sigma_k}^2}\right)\right)$$

else if $E \leq E_g$:

$$\varepsilon_i(E) = \frac{{E_p}^2E_\Gamma}{E(E^2 + {E_\Gamma}^2)} + \sum_j\left(\frac{f_j(E\Gamma_j)}{\left((E_j^{center})^2 - E^2\right)^2 + \left(E\Gamma_j\right)^2}\right) + \sum_k\left(B_k\, exp\left(\frac{(E - E_k^G)^2}{2{\sigma_k}^2}\right)\right)$$

where E_g corresponds to the band gap energy. A, E_0, C and E_g are fitted parameters of the Tauc-Lorentz law. E_p and E_Γ, are fitted parameters of the Drude law (related to plasma frequency and damping rate, respectively). Γ_j, E_j^{center}, and f_j are fitted parameters of the individual Lorentz oscillators (related to broadening, peak position and amplitude respectively). B_k, E_k^G, and σ_k are fitted parameters of the individual Gaussian oscillators (related to amplitude, peak position and broadening respectively).

The real part of the complex dielectric function is then acquired via the Kramers-Kronig relations[3]:

$$\varepsilon_r(E) = \varepsilon_\infty + \frac{2}{\pi} P \int_0^\infty \frac{\xi \varepsilon_i(\xi)}{\xi^2 - E^2} d\xi$$

Where P denotes the Cauchy principal value to handle singularity when $\xi^2 = E^2$.

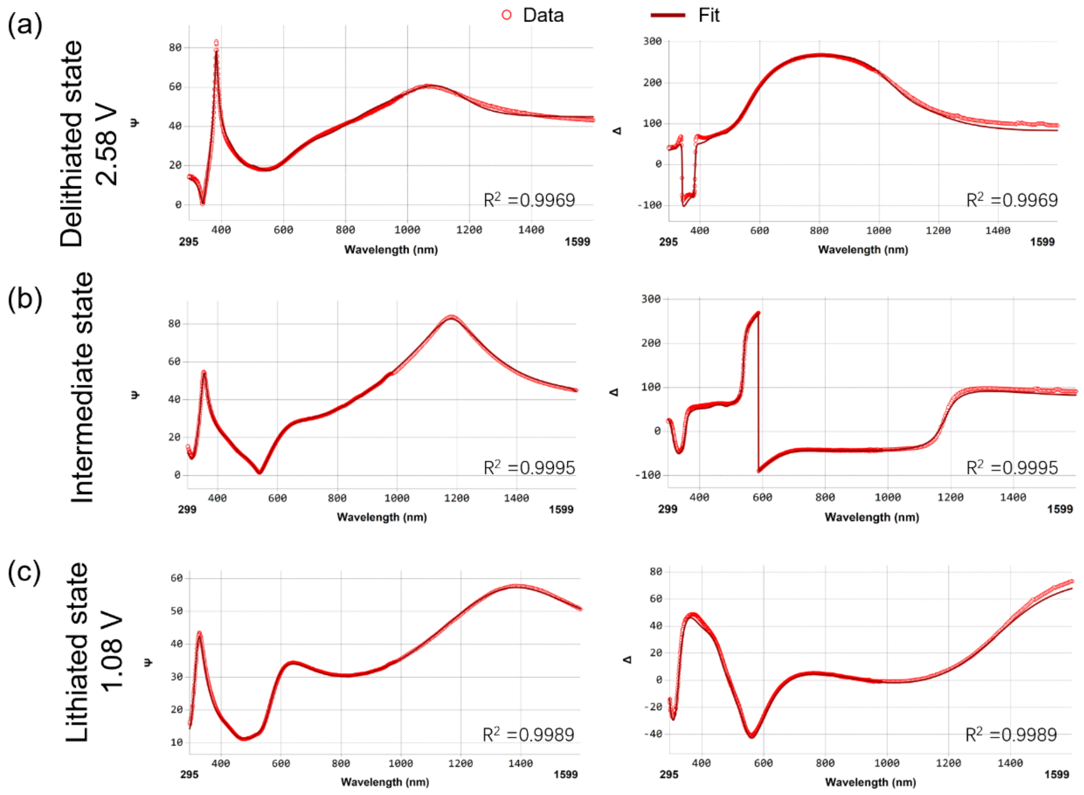

Figure S5: Example fitted Ψ and Δ ellipsometry data at (a) the delithiated TiO_2-anatase state at 2.58 V, (b) an intermediate state and (c) the lithiated state at 1.08 V.

A combination of global search, semi-global and local search algorithms were utilized to ensure that the best overall fit is achieved without getting stuck in local solution. This combination uses six passes with different regression fitting algorithms: Grid search, Levenberg Marquardt algorithm (LMA), Simplex, and Price's algorithm.

The Direct grid searching algorithm[4] is a brute-force method which examines all the predefined grid points in the decision space by evaluating the values of the figure of merit function, thus enabling a global search independent of initial parameters. The weighted Price's algorithm[5] is a controlled random search method (it combines pure random search and clustering strategies). On the other hand, the Nelder-Mead simplex algorithm[6] and the LMA are[7] local searches, speeding up computational time with rapid convergence when the initial parameters are already optimized to be near the global minimum.

Each pass optimizes different parameters of the dispersion laws/oscillators, as detailed in Supporting Table S1. Each measurement data point (Psi-Delta spectra in time) was fitted independently with this algorithm to ensure reliable regression (goodness of fit R^2>0.995).

Table S1: Order and dispersion laws/oscillators fit during regression fitting of raw ellipsometry data. The Grid search is a global fitting algorithm, Price's is a semi-global, and Levenberg Marquardt's algorithm (LMA) is a local search.

**Pass**	**1st Grid Search**	**2nd LMA**	**3rd Simplex**	**4th LMA**	**5th Price's**	**6th LMA**
Layer Thickness		✓	✓	✓		✓
Tauc-Lorentz	✓	✓	✓	✓		✓
Drude		✓	✓			
Lorentz1	✓	✓		✓	✓	✓
Lorentz2		✓		✓		✓
Gauss1		✓		✓		✓
Gauss2		✓	✓	✓	✓	✓
Gauss3		✓		✓		✓

3. Parameters Extracted from *Operando* Ellipsometry

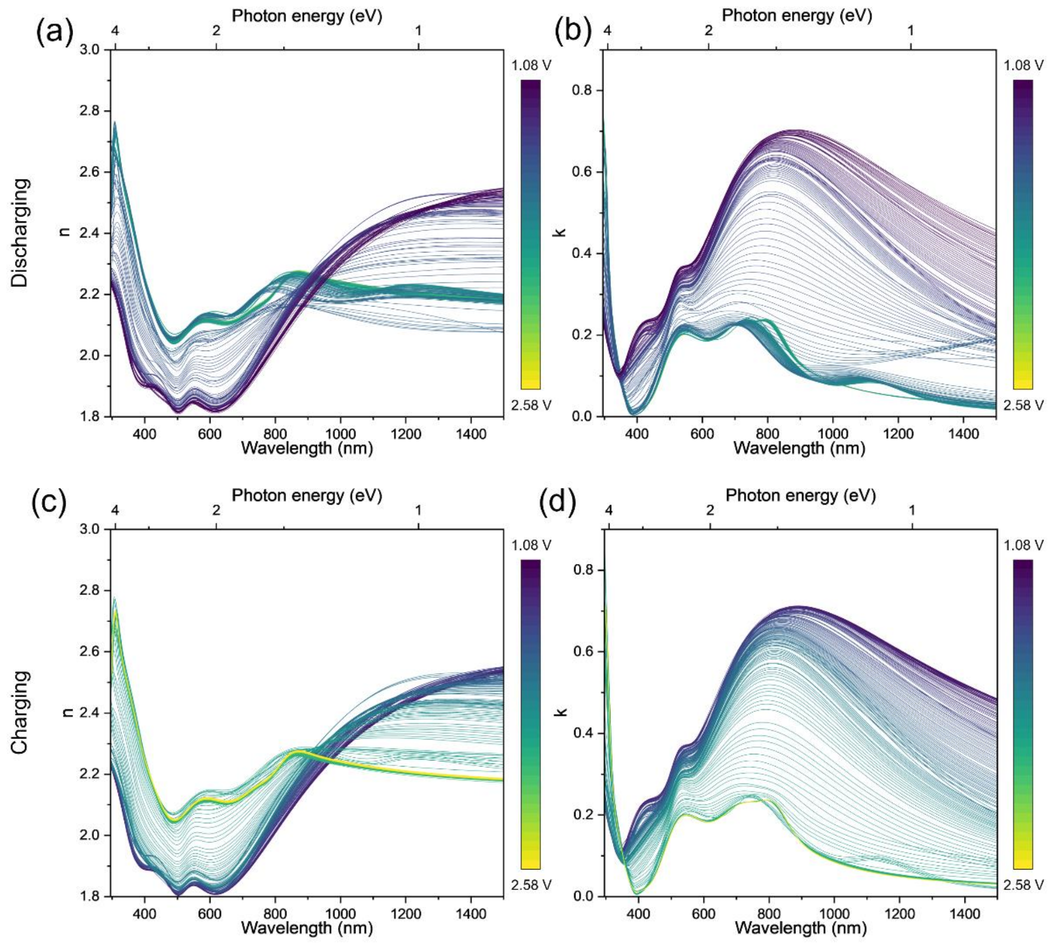

Figure S6: Evolution of refractive index (n) and extinction coefficient (k) during cycling. (a) n and (b) k during discharge, plus (c) n and (d) k during charging.

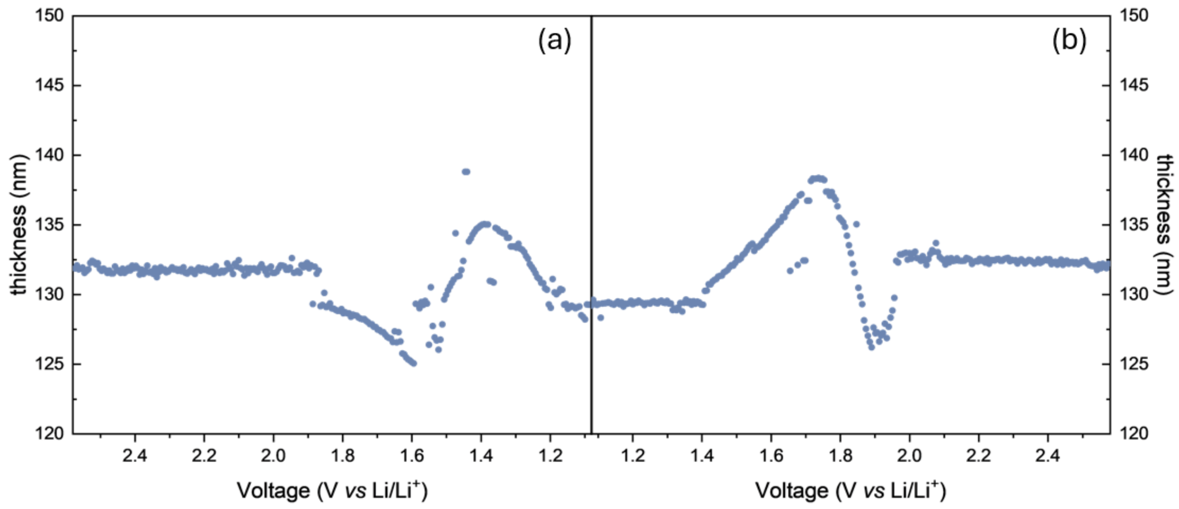

Figure S7: Evolution of film thickness during cycling at 0.1 mV s-1. (a) Thickness change during lithiation. (b) Thickness change during de-lithiation

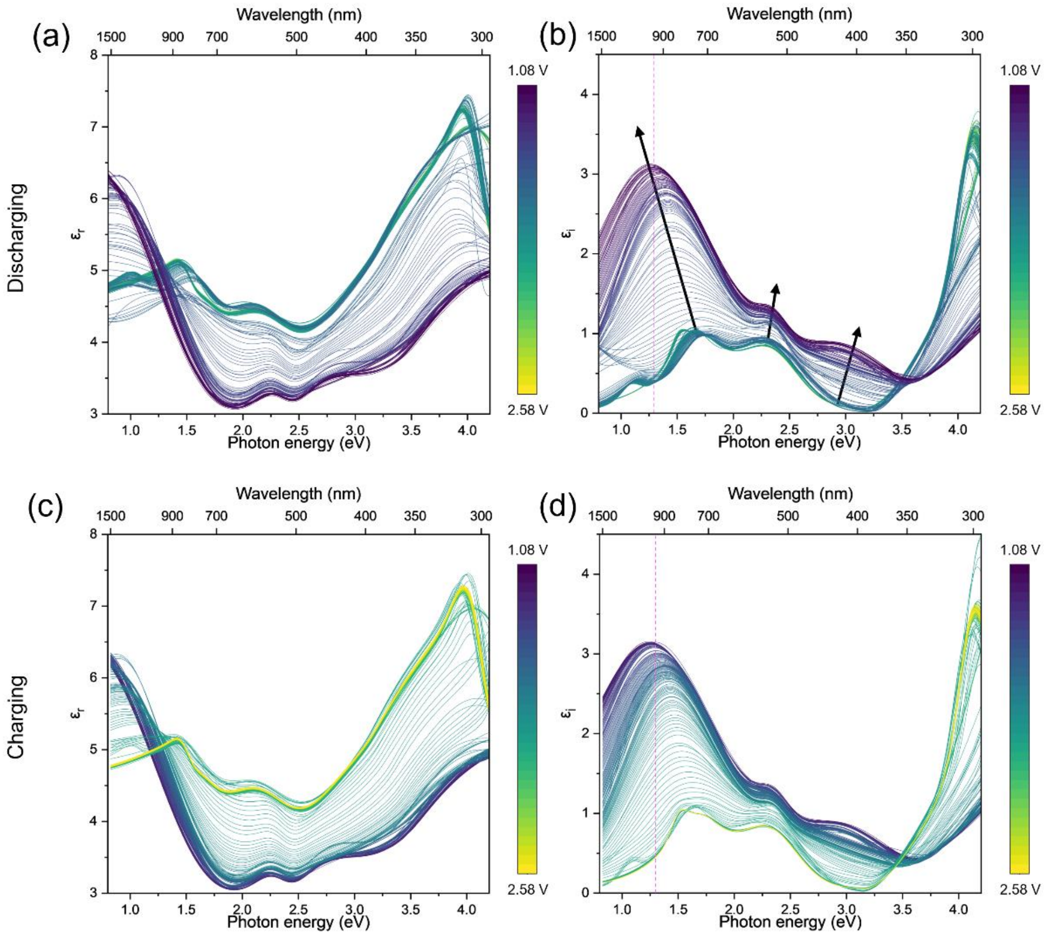

.

Figure S8: Evolution of real (ε_r) and imaginary (ε_i) components of the dielectric constant during electrochemical lithiation of a TiO_2-anatase film. (a) ε_r and (b) ε_i during discharge, plus (c) ε_r and (d) ε_i during charging. Note, ε_i may be used to monitor charge in the film (Figure S9), which is done at 1.3 eV (pink dashed lines, (c,d). However, features in ε_i suffer from peak shift (examples of which are labelled in Figure (b)). Thus, the photon energy must be carefully chosen otherwise information about the system may be lost.

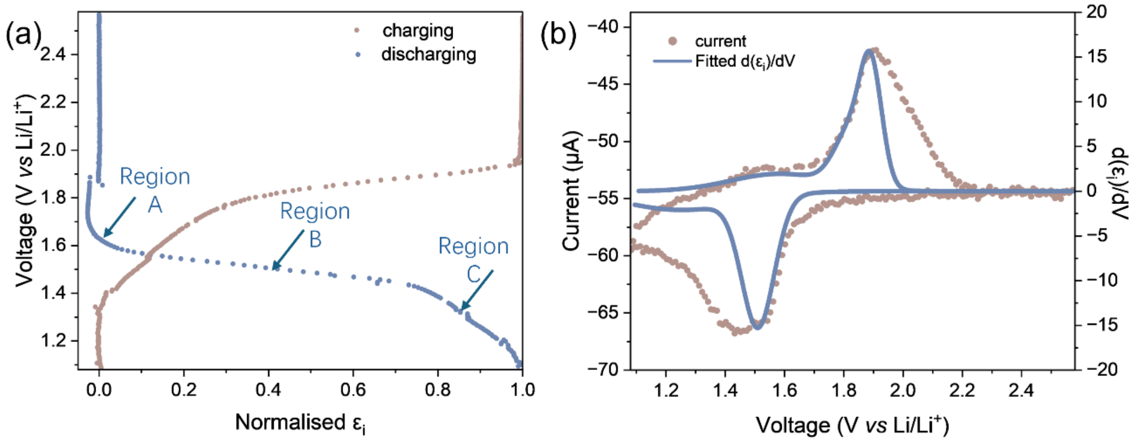

Figure S9: (a) Opto-charge-discharge curve and (b) opto-voltammogram determined from monitoring the evolution of ε_i at 1.3 eV (pink dashed line, Figure S8 b&d).

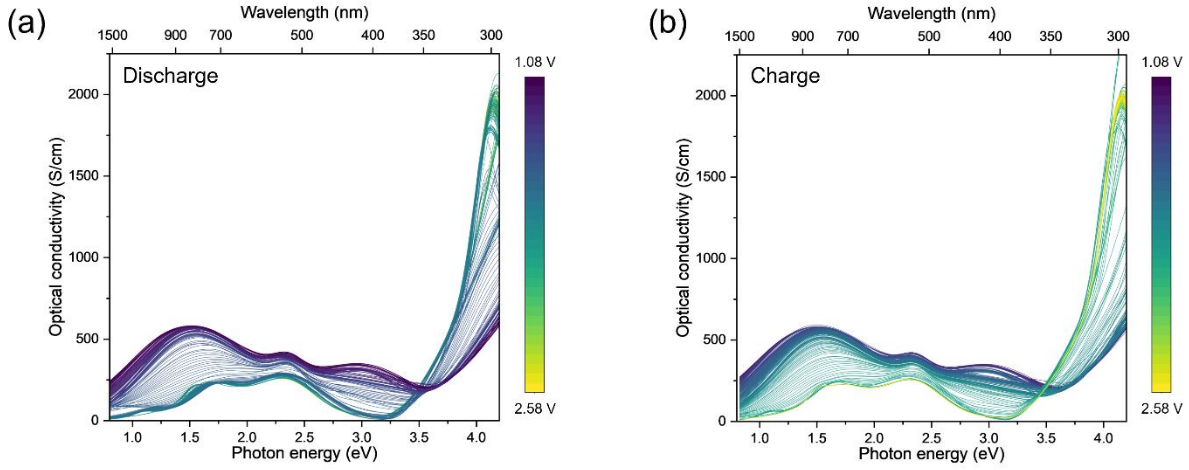

Figure S10: Evolution of optical conductivity of TiO_2 anatase thin film during (a) discharge and (b) charge. Optical conductivity is given by: $\sigma_{optical} = \frac{4\pi nk}{\lambda Z} = \frac{2\pi\epsilon_i}{\lambda Z}$, $Z = \sqrt{\frac{\mu_0}{\epsilon_o}}$. Like ε_i, features show peak shift.

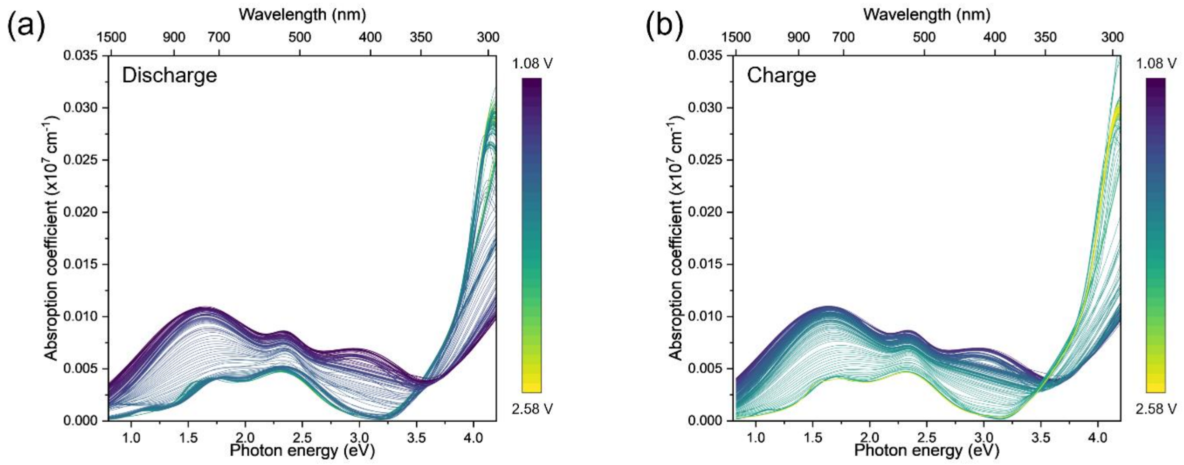

Figure S11: Evolution of the absorption coefficient of a TiO_2 anatase thin film during (a) discharge and (b) charge. The absorption coefficient is given by: $\alpha = \frac{4\pi k}{\lambda} = \frac{2\pi \epsilon_i}{n\lambda}$ which, like ε_i, show peak shift due to ε_i dependence.

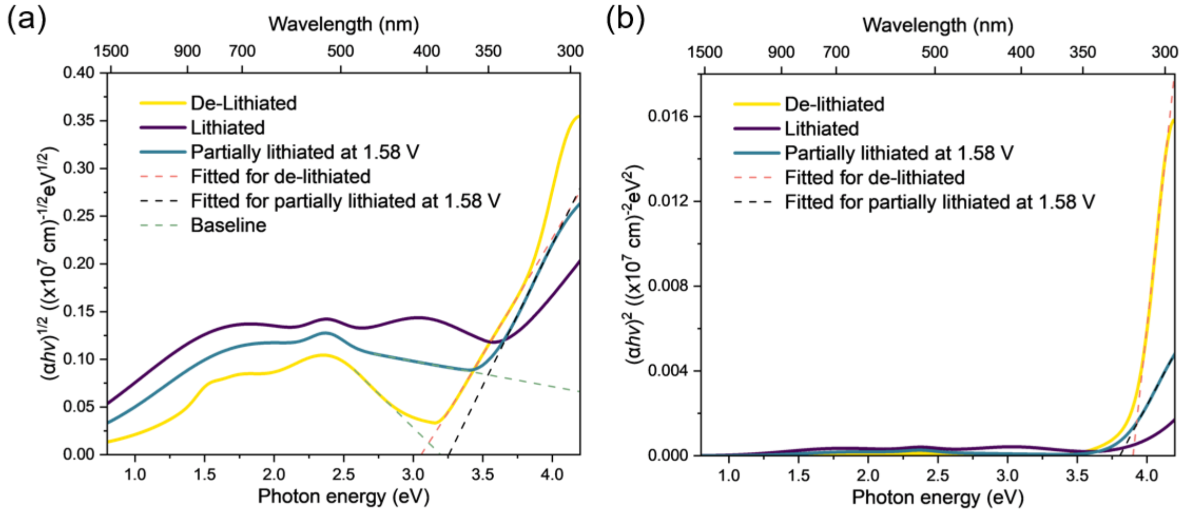

Figure S12: Tauc plots of TiO_2-anatase at different lithiation states, used to extract the optical band gaps. (a) The plot of $(\alpha h\nu)^{1/2}$ *vs.* photon energy corresponds to the indirect band gap. The band gap energy is estimated from the intersection between the linear fit of the absorption edge and the baseline.[8] For de-lithiated TiO_2-anatase, the band gap is approximately 3.15 eV, increasing to around 3.55 eV upon partial lithiation at 1.58 V vs. Li/Li^+. (b) The plot of $(\alpha h\nu)^2$ represents the direct band gap, estimated to be around 3.9 eV for the de-lithiated state and ~3.8 eV for the lithiated state at 1.58 V. Note that in the lithiated state, the full determination of the band edge is limited by the photon energy range of the measurement.

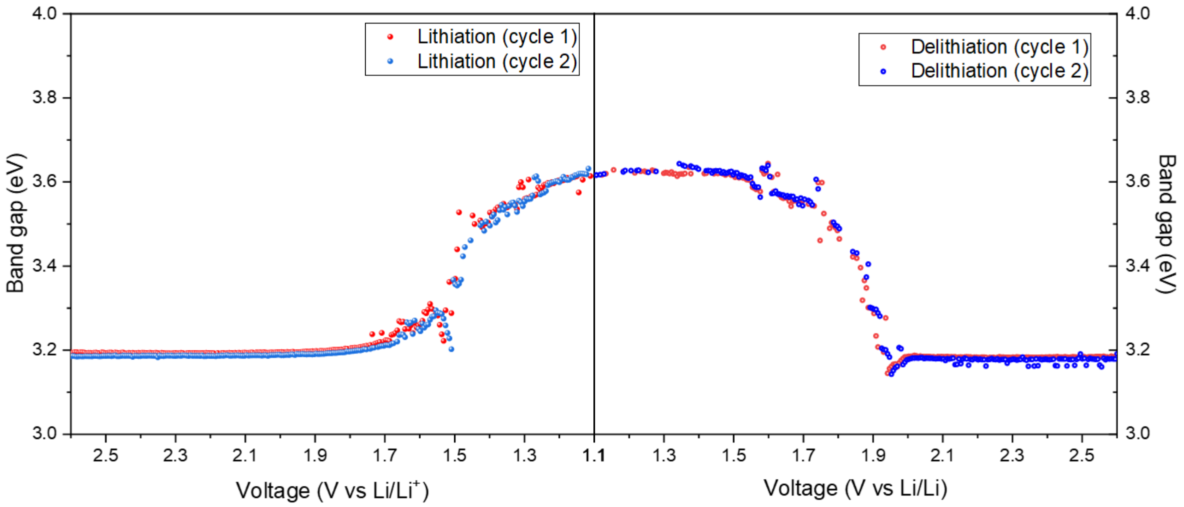

Figure S13: Evolution of indirect band gap energy during cycling at 0.1 mV s^{-1}. (a) Band gap energy during lithiation. (b) Band gap energy during de-lithiation.

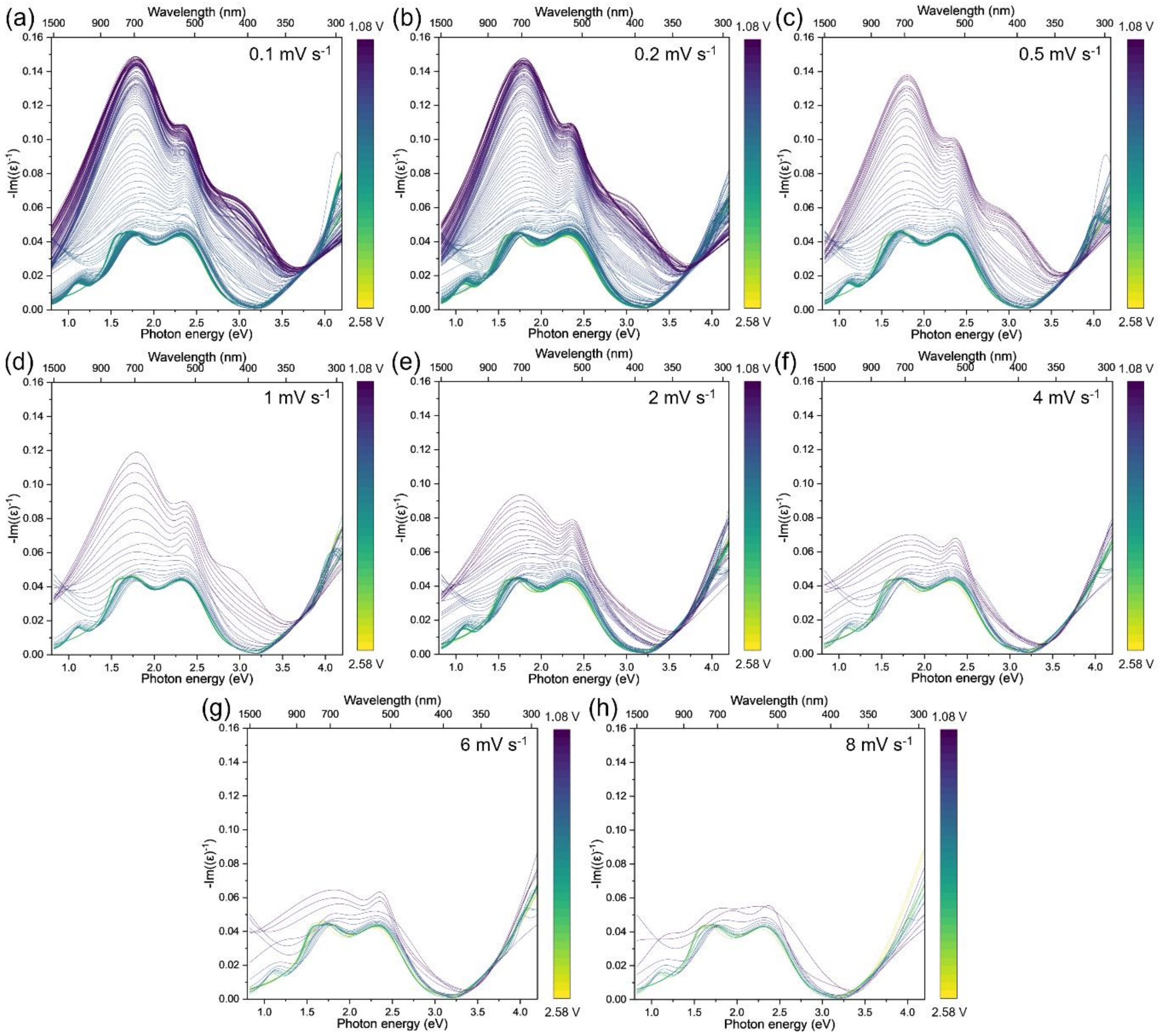

Figure S14: Evolution of the ELF under different CV scan rates during discharge.

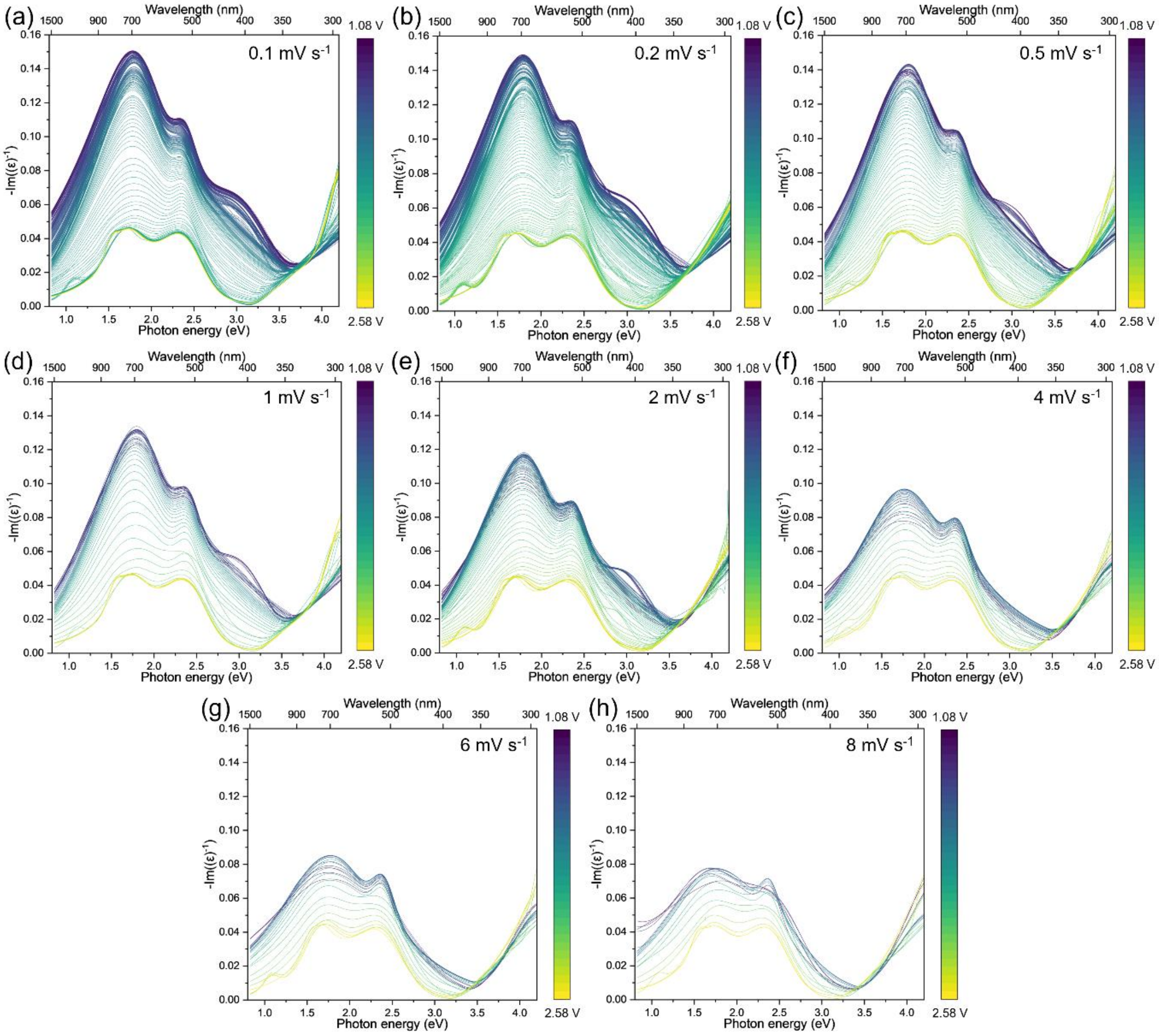

Figure S15: Evolution of the ELF under different CV scan rates during charging.

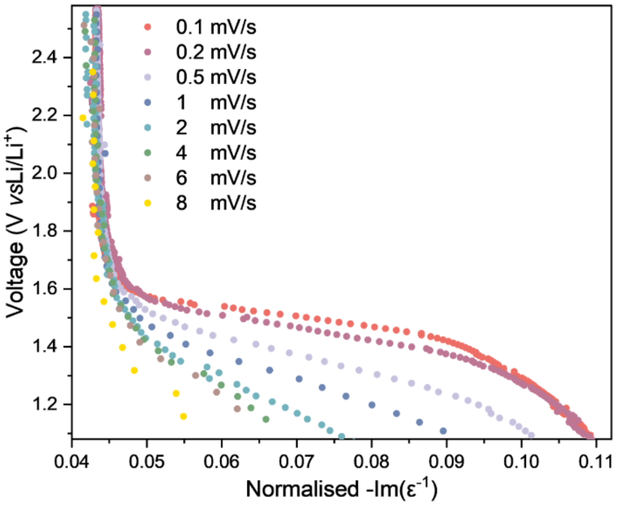

Figure S16: Normalised opto-charge curve (2.3 eV) at various CV scan rates (0.1-8 mV s^{-1})

Supporting References

(1) Gentili, V.; Brutti, S.; Hardwick, L. J.; Armstrong, A. R.; Panero, S.; Bruce, P. G. Lithium Insertion into Anatase Nanotubes. *Chemistry of Materials,* **2012**, *24* (22), 4468–4476. https://doi.org/10.1021/CM302912F.

(2) Bhatia, A.; Hallot, M.; Leviel, C.; Roussel, P.; Pereira-Ramos, J. P.; Lethien, C.; Baddour-Hadjean, R. Electrochemical Lithium Insertion into TiO2 Anatase ALD Thin Films for Li-Ion Microbatteries: An Atomic-Scale Picture Provided by Raman Spectroscopy. *Adv Mater Interfaces* **2023**, *10* (10), 2202141. https://doi.org/10.1002/ADMI.202202141.

(3) H. Fujiwara. *Spectroscopic Ellipsometry: Principles and Applications*. John Wiley & Sons. **2007**

(4) Allgaier, G.; Huelsman, L. A Grid Search Optimization Subroutine for Use with the GOSPEL Optimization Software Package. **1969**.

(5) Brachetti, P.; De Felice Ciccoli, M.; Di Pillo, G.; Lucidi, S. A New Version of the Price's Algorithm for Global Optimization. *Journal of Global Optimization* **1997**, *10* (2), 165–184. https://doi.org/10.1023/A:1008250020656.

(6) Nelder, J. A.; Mead, R. A Simplex Method for Function Minimization. *Comput J.* **1965**, *7* (4), 308–313. https://doi.org/10.1093/COMJNL/7.4.308.

(7) Marquardt, D. W. An Algorithm for Least-Squares Estimation of Nonlinear Parameters. *Journal of the Society for Industrial and Applied Mathematics* **1963**, *11* (2), 431–441. https://doi.org/10.1137/0111030.

(8) Makuła, P.; Pacia, M.; Macyk, W. How To Correctly Determine the Band Gap Energy of Modified Semiconductor Photocatalysts Based on UV-Vis Spectra. *Journal of Physical Chemistry Letters* **2018**, *9* (23), 6814–6817. https://doi.org/10.1021/ACS.JPCLETT.8B02892.
